# Predicting the risk of malaria re-introduction in countries certified malaria-free: a systematic review

**DOI:** 10.1186/s12936-023-04604-4

**Published:** 2023-06-06

**Authors:** Guangyu Lu, Dongying Zhang, Juan Chen, Yuanyuan Cao, Liying Chai, Kaixuan Liu, Zeying Chong, Yuying Zhang, Yan Lu, Anna-Katharina Heuschen, Olaf Müller, Guoding Zhu, Jun Cao

**Affiliations:** 1 School of Public Health, Medical College of Yangzhou University, Yangzhou University, Yangzhou, 225007 China; 2 Jiangsu Key Laboratory of Zoonosis, Yangzhou, China; 3 Center for Global Health, School of Public Health, Nanjing Medical University, Nanjing, China; 4 School of Nursing, Medical College of Yangzhou University, Yangzhou University, Yangzhou, China; 5 National Health Commission Key Laboratory of Parasitic Disease Control and Prevention, Jiangsu Provincial Key Laboratory On Parasite and Vector Control Technology, Jiangsu Institute of Parasitic Diseases, Wuxi, China; 6 Nanjing Health and Customs Quarantine Office, Nanjing, China; 7 Institute of Global Health, Medical School, Ruprecht-Karls-University, Heidelberg, Germany

**Keywords:** Malaria elimination, Re-introduction risk, Surveillance, Prediction models, Systematic review

## Abstract

**Background:**

Predicting the risk of malaria in countries certified malaria-free is crucial for the prevention of re-introduction. This review aimed to identify and describe existing prediction models for malaria re-introduction risk in eliminated settings.

**Methods:**

A systematic literature search following the PRISMA guidelines was carried out. Studies that developed or validated a malaria risk prediction model in eliminated settings were included. At least two authors independently extracted data using a pre-defined checklist developed by experts in the field. The risk of bias was assessed using both the prediction model risk of bias assessment tool (PROBAST) and the adapted Newcastle–Ottawa Scale (aNOS).

**Results:**

A total 10,075 references were screened and 10 articles describing 11 malaria re-introduction risk prediction models in 6 countries certified malaria free. Three-fifths of the included prediction models were developed for the European region. Identified parameters predicting malaria re-introduction risk included environmental and meteorological, vectorial, population migration, and surveillance and response related factors. Substantial heterogeneity in predictors was observed among the models. All studies were rated at a high risk of bias by PROBAST, mostly because of a lack of internal and external validation of the models. Some studies were rated at a low risk of bias by the aNOS scale.

**Conclusions:**

Malaria re-introduction risk remains substantial in many countries that have eliminated malaria. Multiple factors were identified which could predict malaria risk in eliminated settings. Although the population movement is well acknowledged as a risk factor associated with the malaria re-introduction risk in eliminated settings, it is not frequently incorporated in the risk prediction models. This review indicated that the proposed models were generally poorly validated. Therefore, future emphasis should be first placed on the validation of existing models.

**Supplementary Information:**

The online version contains supplementary material available at 10.1186/s12936-023-04604-4.

## Background

Despite the negative impact of the COVID-19 pandemic, there is progress toward malaria elimination. In 2021, 84 malaria-endemic countries compared to 108 in 2000 were identified [1]. Between 2000 and 2020, 23 countries were declared malaria-free based on zero indigenous malaria cases reported in 3 consecutive years [2]. In 2010–2021, total malaria cases in the E-2025 countries (malaria-eliminating countries for 2025) reduced by 82.8%, demonstrating continued efforts by countries toward their elimination goals [1]. With accelerated progress toward eliminating malaria in recent decades, the main concern is its re-emergence in areas where this disease was previously eliminated. Re-emergence can be facilitated through population movement from endemic countries, particularly due to the presence of competent vectors and favourable climatic conditions [3]. In recent years, reintroduced autochthonous malaria cases have even been sporadically reported from Italy, France, Spain and Greece [4–8].

Surveillance is a core and an effective intervention to support malaria elimination goal. The importation of parasites to an area with competent vectors makes the human population susceptible to risk. Thus, the rate of immigration of infected individuals and the prevalence of mosquito vectors are usually the focus of surveillance [3, 9–12]. Moreover, meteorological conditions, including local temperatures, rainfall, and humidity, are frequently considered for predicting the risk of malaria re-introduction, as these strongly affect the life cycle and survival of parasites and vectors [13–17]. However, the risk of malaria re-introduction in eliminated settings depends on several factors; for example, the reduction of funding for malaria control programmes following successful elimination, inadequate awareness about the possibilities of parasite re-introduction to malaria-free regions, and socioeconomic parameters [13, 18].

Winfried Schröder and Schmidt [19] established a malaria prediction model based on vector capacity and meteorological variables for northwestern Germany, where malaria was eradicated in the early 1950s. Linard et al. [20] assessed the risk of malaria re-emergence in southern France. Sainz-Elipe et al. [21] evaluated the transmission risk in Spain using the gradient model risk index. Romi et al*.* [22] assessed the risk of malaria re-introduction in central Italy through a multifactorial approach. Ranjbar et al*.* [9] predicted the risk of malaria re-introduction in two provinces in Iran. Such prediction models are crucial to facilitate the prioritization of allocation of the health system’s resources and take necessary action promptly to prevent the resurgence of malaria [9]. However, an up-to-date review of existing malaria re-introduction risk models in eliminated settings at a global level is lacking. Therefore, this study aimed to systematically review and critically appraise existing prediction models for malaria re-introduction risk in countries certified malaria-free.

## Methods

This systematic review followed the Preferred Reporting Items for Systematic Reviews and Meta-Analyses (PRISMA) statement guidelines [23]. The protocol was registered on the international prospective register of systematic reviews (PROSPERO) database (CRD42022381245).

### Search strategy

A literature search was performed in PubMed, Web of Science, Cochrane Library, China National Knowledge Infrastructure (CNKI), and reference lists of publications by using the following key words: “*malaria”*, “*Malaria, Vivax*”, “*Malaria, Falciparum*”, “*acute malaria*”, “predict*”, “predictive model”, “prediction model”, “risk prediction”, “risk score”, “risk calculation”, and “risk assessment” (Additional file 1: Appendix 1). This study initially searched databases on 01 June 2022, with an update on 16th March 2023. Citations of relevant articles were manually screened to identify additional studies.

### Eligibility criteria

Studies that developed or validated prediction models for malaria re-introduction risk in countries that are already certifed as malaria-free, regardless of their design. Studies published in English or Chinese languages were included. The prediction rule was defined based on the combination of three or more risk factors. Studies that only analysed individual risk factors influencing malaria re-introduction without establishing prediction models were excluded. If more than two articles described one prediction model, the model was recorded once, and the information was extracted fully from all the relevant articles.

### Data extraction and analysis

After eliminating duplicate entries, three researchers analyzed the titles and abstracts to confirm that all articles met the inclusion criteria and record the reasons for exclusion. Papers excluded for specific reasons were recorded. All studies were evaluated independently by at least two investigators. For potential relevant studies, the full text was obtained, and two investigators (JC and DYZ) independently assessed its eligibility. All data were independently characterized following a standardized protocol, including the title, first author name, year of publication, study location, source of data (surveillance data or cross-sectional survey), predictive variables in the established model, performance of the model (sensitivity and specificity), the internal and external validity of the established prediction model, and limitations of the model. Disagreements were resolved by consensus or through a discussion with a third reviewer. Considering the high heterogeneity in the prediction model, the limitations of the included studies were analyzed by thematic content analysis.

### Quality assessment

Considering that the prediction model risk of bias assessment tool (PROBAST) is well acknowledged for a quality appraisal of prediction models, it was adopted to assess their risk of bias and applicability by the two independent reviewers. Moreover, the adapted Newcastle–Ottawa Scale (aNOS) scale was adapted to appraise the risk of bias in the included studies, as these were mainly observational [24, 25]. PROBAST consisted of 20 items and included the specifics about the research design, study population, outcome of the model, predictors, handling of the data, and performance measures (Additional file 1: Appendix 2). The aNOS scale included an assessment of the sample selection (4 criteria), comparability (1 criterion), and outcomes (2 criteria). Scores on this instrument range from 1 to 10, with higher scores indicating higher quality (Additional file 1: Appendix 3). Any disagreements were resolved by mutual agreement.

## Results

**Fig. 1:**
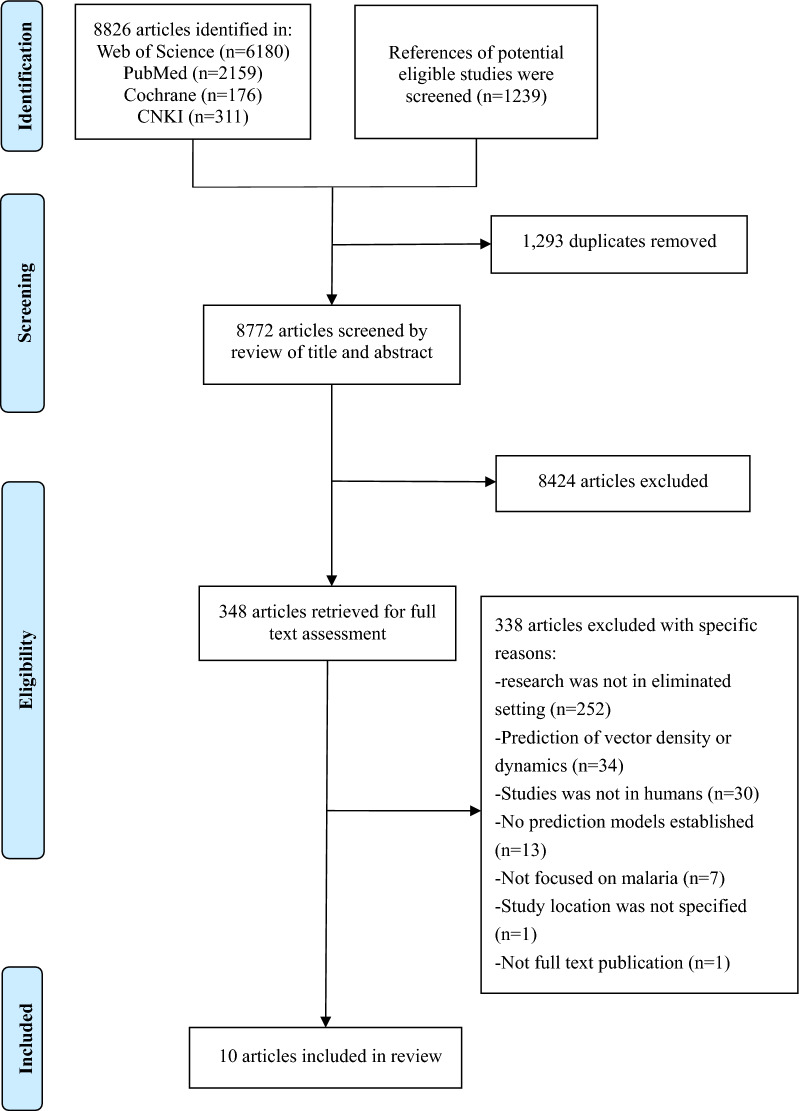
A summary flow of study selection process

A total of 8772 articles were identified after the electronic search of the databases and the removal of duplicates. The titles and abstracts of these articles were screened and 348 articles were subjected to full-text review (Fig. 1). Of these studies, 338 were excluded after reading their full texts because these did not fulfill the inclusion criteria (Additional file 1: Appendix 4). Finally, 10 studies were included in the analysis [19, 21, 22, 26–32].

### General characteristics of the included studies

The general characteristics of the 10 articles reporting 11 malaria re-introduction risk prediction models in malaria eliminated settings were summarized in Table 1. Among all included prediction models, seven were developed for the European region (Germany, UK, Spain, Italy, and Greece), and four for China. The included studies were published between 2008 and 2023, with four (4/10, 40%) being published after 2020.

**Table 1:** Study characteristics and summary of the 11 prediction models reported in 10 included the studies

No	Author, year	Study location and country	Data source	Malaria epidemiology in study place	Methods used in prediction modelling	Variables included in the model					The performance of the model	The external validation
						Environmental and meteorological factors	Vectorial factors	Population migration	Surveillance and response related factors	Others		
**1**	Schöder and Schmidt 2008	Lower Saxony, Germany	Routine surveillance data and literature record	Malaria was eliminated in 1964 Exisitng Vector: *Anopheles atroparvus*	Mathematical method: Basic reproduction number (*R*_*0*_)	Temperature (average air temperature)	1.Mosquito density; 2.The number of bites per person per day; 3.The length of the gonotrophic cycle; 4.Proportion of female mosquitoes with developing parasites after taking an infected blood meal; 5.Daily survival probability of an adult female mosquito; 6.Period of parasite development in the adult female mosquito in days	–	–	The immunity of local people	Not mentioned	Not mentioned
2	Lindsay et al. 2010	The United Kingdom	Routine surveillance data	Malaria was eliminated in 1963 Exisitng Vector *Anopheles atroparvus*	Mathematical method: Basic reproduction number (*R*_*0*_)	Temperature (average air temperature)	1.Mosquito density; 2.The number of bites per person per day; 3.The length of the gonotrophic cycle; 4.Proportion of female mosquitoes with developing parasites after taking an infected blood meal; 5.Daily survival probability of an adult female mosquito; 6.Period of parasite development in the adult female mosquito in days	Number of imported cases	–	The immunity of local people	Not mentioned	External validation was performed by comparing potential distribution of malaria between 1961–1990 and 1859–1864
					Statistical method: Logistic regression	1.Temperature (the mean temperature of the warmest month; the mean temperature of the coldest month); 2.Index of wetness (an index of wetness which is a measure of potential evaporation in relation to rainfall)	–	Number of imported cases	–	Population density	Not mentioned	Not mentioned
3	Sainz-Elipe et al. 2010	The Ebro Delta in the province of Tarragona, Spain	Routine surveillance data and cross-sectional survey	Malaria was eliminated in 1964 Exisitng Vector: *Anopheles atroparvus*	Mathematical method: Gradient Model Risk Index	1.Temperature (mean maximum temperature, mean minimum temperature, mean environmental temperature); 2.Rainfall; 3.Relative humidity; 4.Potential evapotranspiration; 5.Wind speed; 6.Vapor pressure; 7.Global radiation; 8.Terrain characterization	Population dynamics of *A. atroparvus*	–	–		Not mentioned	Not mentioned
4	Romi et al. 2012	Maremma plain,central Italy	Routine surveillance data and cross-sectional survey	Malaria was eliminated in 1970 Exisitng Vector: *Anopheles maculipennis*	Mathematical method: Receptivity of the area-Susceptibility of the vector-Vulnerability of the territory	1.Rainfall; 2.Potential evapotranspiration	1.Species, blood meal source and age population structure of mosquito; 2.Abundance of larvae/adult mosquito; 3.The length of the possible transmission season for *P. vivax* and *P. falciparum*; 4.The vectorial capacity and host feeding preference of *An. labranchiae*; 5.Vectorial capacity of *An. labranchiae* in the site where the species is most abundant; 6.The possibility of vector may feed on gametocyte carriers	–	–	–	Not mentioned	Not mentioned
5	Sudre et al. 2013	Greece	Routine surveillance data	Malaria was eliminated in 2012 Exisitng Vector *Anopheles* sacharovi; *Anopheles* superpictus	Statistical method: Bootstrap model	1.Temperature; 2.Vegetation seasonal variations; 3.Altitude; 4.Land-cover categories	–	–	–	Population density	Sensitivity:0.98, Specificity:0.98	Not mentioned
6	Pergantas et al. 2017	8 municipalities of the Prefecture of central Greece	From the European Environment and Epidemiology Network data repository	Malaria was eliminated in 2012 Exisitng Vector *Anopheles* sacharovi; *Anopheles* superpictus	Mathematical method: Basic reproduction number (*R*_*0*_)	Temperature	1.The ratio of mosquitoes/humans; 2.The biting rate(proportion of mosquitoes that feed on humans each day); 3.The mosquito latent period (the number of days from infection to infectiousness); 4.Mosquito death rate per day; 5.Probability a bite produces infection to a human; 6.Probability a mosquito becomes infected after biting an infected human	1.Migrant; 2.Distances of migrant population from the larvae areas	–	The immunity of local people	Not mentioned	Not mentioned
7	Kamana et al. 2022	China	Routine surveillance data	Malaria was eliminated in 2021 Exisitng Vector: *Anopheles sinensis, Anopheles minimus, Anopheles dirus,* *Anopheles lesteri*	Machine learning method: LSTM neural networks	1.Temperature (average maximum and minimum temperature); 2.Rainfall; 3.Relative humidity	–	–	–		Average prediction accuracy of LSTMSeq2Seq model = 87.3%	Not mentioned
8	Lan et al. 2022	China	Cross-sectional survey	Malaria was eliminated in 2021 Exisitng Vector *Anopheles sinensis, Anopheles minimus, Anopheles dirus,* *Anopheles lesteri*	Delphi method	1.Temperature; 2.Rainfall; 3.Altitude	1.Type of vector; 2.Density of vector; 3.Biting rate; 4.Susceptibility of vectors to plasmodium 5. Sensitivity of vectors to insecticides	1.Number of imported case; 2.Companion of imported cases	1.Awareness of malaria control knowledge; 2.Multi-sectoral joint prevention and control mechanism; 3.Implementation of ' 1–3-7 malaria surveillance and response strategy 4.Vector surveillance 5.Diagnostic capability 6.Proportion of initial diagnosis of malaria by medical institutions; 7.Proportion of fever patients receiving blood tests	1.Time of malaria cases imported to China; 2.Malaria prevalence in the overseas country	Not mentioned	Not mentioned
9	Li et al. 2022	Changsha, Hunan Province, China	Routine surveillance data	Malaria was eliminated in 2021 Exisitng Vector: *Anopheles sinensis, Anopheles minimus, Anopheles dirus,* *Anopheles lesteri*	Delphi method	–	Anopheles mosquito-borne malaria risk index (Anopheles species)	1.Number of imported cases; 2.Type of imported malaria cases	1.Implementation of ' 1–3-7 malaria surveillance and response strategy 2.Capability of blood testing (laboratory diagnosis rate); 3. Standardized treatment rate; 4.Treatment capacity of medical institutions	Funding for malaria prevention and control	Not mentioned	Not mentioned
10	Liu et al. 2023	China	Cross-sectional survey	Malaria was eliminated in 2021 Exisitng Vector: *Anopheles sinensis, Anopheles minimus, Anopheles dirus,* *Anopheles lesteri*	Delphi method	1.Temperature; 2.Rainfall; 3.Altitude 4. Relative humidity 5. Season 6. Anopheles breeding environment 7. Livestock breeding	1.Local Anopheles species 2.Anopheles density 3.Sensitivity of vectors to insecticides 4.Vectorial capacity 5.Susceptibility of vectors to plasmodium	1.Number of migrants 2.Awareness of malaria control knowledge among migrants 3.Mosquito bites of imported personnel in the overseas country 4.Occupation in the overseas country 5.Residence time in the overseas country 6.Residency in the overseas country (e.g. urban/rural/estate) 7.Number of imported cases	1.Diagnostic and discovery capability of customs district 2.Awareness of health-seeking of imported personnel 3.Diagnostic and treatment capacity of health workers 4.The number of malaria control staff 5.stockpiling anti-malarial drugs 6.Implementation of ' 1–3-7 malaria surveillance and response strategy 7.Vector surveillance 8.Multi-sectoral joint prevention and control mechanism	1.Malaria surveillence capacity in countries where malaria has been imported 2.Accessibility of antimalarial drugs in the overseas country 3.Governments ' attention and financial support 4.The geography and malaria situation in countries where malaria has been imported (e.g. whether the country is cross-border or not) 5.The number of years after malaria elimination 6.Plasmodium species in the overseas country	Not mentioned	Not mentioned

### The methodology used in prediction models

Nearly half of the models (5/11, 45.6%) were developed by statistical and mathematical methods, followed by machining learning (3/11, 27.3%) and Delphi method (3/11, 27.3%). Of the 11 included malaria prediction models, the majority were developed based on routine surveillance data (8/11, 88.9%).

### Variables included in the prediction model

**Fig. 2:**
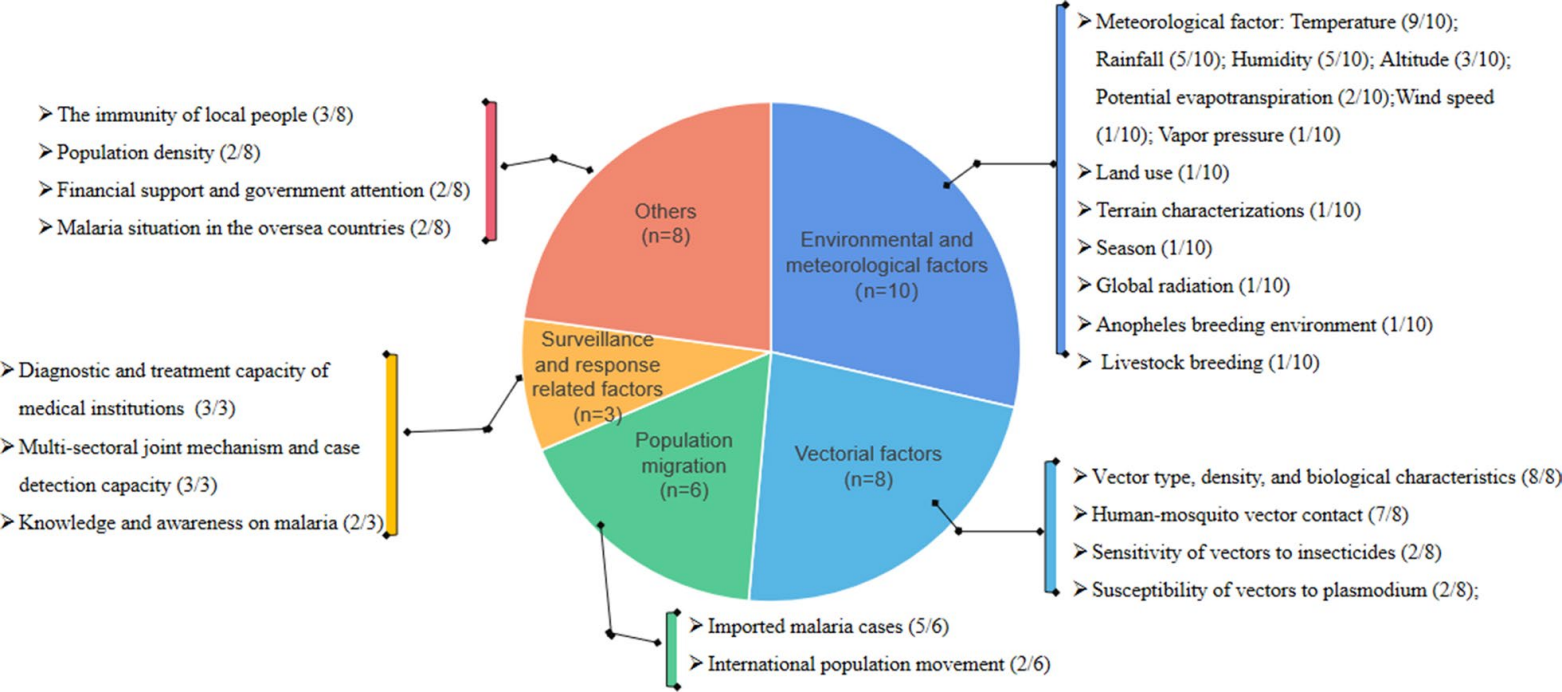
Five domains of predicting factors associated with malaria risk in eliminated settings

Predictors included in the 11 prediction models were identified and classified into five categories, namely environmental and meteorological, vectorial, population migration, surveillance and response related factors, and other factors (Fig. 2).

### *Environmental and meteorological factors*

Environmental and meteorological factors were included in 10 models. Temperature parameters (9/10, 90%), rainfall (5/10, 50%), humidity (5/10, 50%) and altitude (3/10, 30%) were most frequently incorporated in the prediction models. Land use (1/10, 10%) was incorporated in one model, mainly including vegetation seasonal variations and land-cover categories [28]. Terrain characterizations, wind speed, global radiation, vapour pressure were identified in one model (1/10, 10%) [21].

### *Vectorial factors*

Vector factors were incorporated in eight prediction models. The most commonly included vectorial factors identified included vectorial suitability (a combination of vector type, density, and biological characteristics) (8/8, 100%), human-vector contact (number of bites per person per day) (7/8, 87.5%), sensitivity of vectors to insecticides (2/8, 25%), and susceptibility of vectors to *Plasmodium* (2/8, 25%).

### *Population migration*

Population migration was considered a predictor in six models. The socioeconomic status and epidemiological characteristics of imported cases of malaria were particularly interesting factors considered in predicting malaria re-introduction risk in countries certified malaria free and prevention of the re-introduction of malaria (5/6, 83.3%). Moreover, measurements of international population movement from malaria-endemic regions were considered in two models (33.3%).

#### *Surveillance and response related factors*

Surveillance and response related factors were considered in three models. Of which, diagnostic and treatment capacity was frequently incorporated, reflected by the capacity of medical institutions and stockpiling anti-malarial drugs. Moreover, researchers have acknowledged the capacity of cases detection and multi-sector joint mechanism is a significant predictor for malaria re-introduction risk in eliminated settings. Two models have included knowledge and awareness on malaria among the general population as a predictor for the risk of malaria re-introduction.

#### *Others*

Other factors identified here included the immunity of the local people (3/8, 37.5%),financial support and government attention (2/8, 25%), population density (2/8, 25%), and malaria situation in oversea countries (2/8, 25%).

### Performance and validation of the models

Of the 11 prediction models, two studies reported their model performance. Of which, one reported sensitivity and specificity of 98% and 98%, respectively [28], while one reported accuracy of 0.873 [27].

Only one model were externally validated by comparing potential distribution of malaria between 1961–1990 and 1859–1864 [26].

### Limitations of the included studies

Of the 10 included studies, 5 reported their limitations of the established prediction models, which could be summarized into the following five aspects: (1) The model based on the Delphi method had the limitation of subjectivity (3/10, 30%). (2) The variables included in the prediction model were not comprehensive (2/10, 20%). (3) There is missing information in the data used in the model (1/10, 10%). (4) Modelling methods by mathematical approaches were time-consuming (1/10, 10%). (5) Predictive models lacked external validation (1/10, 10%) (Additional file 1: Appendix 5).

#### Quality assessment

All models showed a high risk of bias according to the PROBAST assessment, suggesting that their predictive performance, when used in practice, is probably more limited than expected. In particular, the predictions using the models may further lose reliability if not closely fitted to the local context. Bias was introduced by various sources, as summarized in Additional file 1: Appendix 6. 11 models showed a high risk of bias for the analysis domain, mainly attributed to inappropriate handling of missing data, lack of model performance measure evaluation, and absence of validation. The quality of the included studies was also evaluated using aNOS. Of the 10 included studies, scores on the aNOS scale ranged from three to eight, with nine models scoring seven and above, representing a generally favorable model quality performance. However, this scale only evaluated the aspects of sample selection, sample size, and the assessment of predictor variables (Additional file 1: Appendix 7).

## Discussion

A total of 10 studies was included in this review and 11 prediction models for malaria re-introduction risk were identified in eliminated settings. Predictors mainly include environmental and meteorological, vectorial, population migration, and surveillance and response related factors. Models were mainly developed by statistical and mathematical, machining learning and delphi method. Most of the models were developed for the European region. All prediction models showed a high risk of bias owing to a combination of poor reporting, poor methodological conduct, and a lack of validation. Although published evidence for the routine use of the models in real malaria programmes is lacking, the findings provided a global overview of the existing prediction models and commonly used predictors for malaria risk in eliminated settings.

Approximately one third of the prediction models identified in this review were developed in China. China was certified as a malaria-free country in 2021 after several decades of active control and elimination efforts [33], and consequently, imported malaria cases and receptivity of certain areas posed a challenge to sustaining the success in elimination [34]. In recent years, the proportion of imported malaria cases in China is on the rise, especially those imported from sub-Saharan Africa (SSA), which have increased from 83% in 2017 to 91% in 2019 [35]. With the achievement of the malaria elimination goal in China in 2021, prevention of the re-introduction of malaria has attracted great research interest from scholars and public health workers [35–38]. Different with prediction models established in European countries mainly by statistical and mathematical method, Chinese scholars widely utilized Delphi expert consultation, through which expert opinions in the field could be obtained and integrated.

In countries cetificated malaria-free, malaria re-introduction may occur depending on multiple factors. Vectorial factors are frequently incorporated as indicators in predicting malaria risk in eliminated settings. Mosquito species, relative density, gonotrophic cycle, feeding behaviour, and biting activity (e.g., bites per person per day) are regularly considered during the evaluation of vector capacity. This further demonstrates that monitoring mosquito populations is important in malaria surveillance programmes in eliminated settings. However, ways to maintain high-quality mosquito surveillance to allow mosquito control experts to track exactly where the larval and adult mosquito populations are rising or falling is particularly challenging in eliminated settings, especially in resource-limited regions. Nevertheless, the competence of a malaria vector is strongly affected by climatic and environmental factors, which requires consideration [39]. Certain variables that can play a significant role in the complex dynamic processes of disease spread should be considered, including precipitation, humidity, availability of mosquito breeding sites, land use and land cover, and other ecological factors determining the developmental process of the vector mosquitoes [19, 40]. For example, changes in land use and land cover changes were demonstrated to potentially exert a direct impact on the risk of re-emergence of the disease by affecting mosquito breeding grounds (e.g., the surface of marsh wetlands) and the contact rate between people and mosquitoes [20].

Climatic variables are considered environmental factors for increased risk of malaria because of their impacts on both the incubation rate of *Plasmodium* and mosquito vector activities. Previous researchers have employed a host of environmental factors and meteorological variables, including precipitation, temperature, altitude, and patterns of water availability to create computer models for predicting future malaria transmission [9, 28, 39]. However, the findings in this aspect are inconsistent. For example, elevated temperature was identified as the key meteorological factor correlated with malaria re-emergence in the Huang-huai River region of central China at the beginning of the twenty-first century and the re-emergence of malaria in Greece [29, 41]. However, the increase in temperature was found not to mean an increase the malaria transmission risk, particularly if accompanied by a decrease in precipitation in Spain [21]. Although the temperature is important for parasite development, this indicator should be considered together with the variable related to water availability. Moreover, it is crucial and challenging for scientists to consider all available data and to communicate clearly about the complex interplay between climate and other factors in shaping disease trends [42]. In particular, considering that climate change is itself linked in multiple ways to emerging infectious diseases, global food security and public health [43], and routine surveillance of these predictors serves as an important basis for reemerging vectors and public health preparedness of potential malaria transmission risk of malaria.

In malaria-eliminated settings, malaria re-introduction is also affected by non-climatic factors including migration and human mobility. Previous scholars have emphasized that imported malaria is a particularly important factor and should be considered in the risk evaluation system in countries that have eliminated malaria but have the potency to supports local malaria transmission [44]. However, using such an indicator to predict malaria risk poses challenges. On the one hand, mobile and migrant populations may be underrepresented in routine case data and absent during household visits due to frequent travel [44], i.e., facility-based surveillance approaches may also fail to capture mobile and migrant populations who face barriers to accessing public health facilities, prefer private facilities, or those who do not seek care at all [45]. Moreover, quantifying population movement is not new but a daunting task in many contexts [46]. Travel history surveys, road traffic counts, border crossing questionnaires, shipping schedules, and question in census migration have long been used to obtain data on how people move[47], but each of these data types represents a snapshot of a small area, subpopulation or period, with limits on how much can be inferred beyond the collected data [47]. In addition to quantifying population movement, identifying vulnerable characteristics of migrating populations at high risk of importing malaria could provide an important basis for designing targeted interventions for the prevention of importation and further transmission in case of importation [48].

Malaria surveillance and response capacity are key for evaluation and preparedness for potential re-introduction in eliminated settings. In Europe, despite the substantial number of imported malaria cases and the documented presence of suitable anopheline vectors, autochthonous transmission has not been observed widely, probably due to early diagnosis and treatment afforded by efficient healthcare systems [49]. In contrast, India’s efforts to eliminate malaria have been largely challenged by an acute shortage of health workforce and weak public health surveillance systems [50]. Sri Lanka was once in the malaria pre-elimination phase, but as dichlorodiphenyltrichloroethane (DDT) spraying was discontinued, a disastrous malaria epidemic occurred occurred [18]. Health infrastructure and malaria surveillance capacity were the least commonly incorporated variables in predicting malaria risk in eliminated settings. This may be because of a lack of empirical measurement of competencies to assess public health infrastructure for infectious disease control and prevention, and the lack of easy-to-use instruments to assess the health capacity for infectious disease surveillance. Existing studies have mainly assessed the financial investment, diagnostic capacity of medical staff, diagnostic accuracy based on microscopy of public health workers, and implementation of a training program to reflect the surveillance capacity for malaria. In this regard, a well-acknowledged and user-friendly assessment tool of the surveillance capacity for infectious diseases in eliminated settings is necessitated.

### Challenges to the methodology

The main aim of prediction models for malaria re-introduction risk is to increase preparedness for malaria risk and support public health decision-making. As very limited models were externally validated, it is important to assess the performance of risk prediction models by head-to-head comparison at a global level. The geography, social economy, and malaria epidemiology of the study setting must be carefully described so that the performance of the developed or validated model can be appraised in the given context, and users know which contexts the model applies to when making predictions. However, the included studies in the review mostly lacked an adequate description of their settings (e.g., study population), which leaves users of these models in doubt about the model’s applicability. It is recommend that all future prediction studies improve the description of their regional contexts and modelling choices.

Moreover, instead of developing and updating predictions in the local setting, compiling individual regional data from multiple countries with similar re-introductiom risks and healthcare systems might allow a better understanding of the general ability and implementation of prediction models. This approach could greatly improve the applicability and robustness of prediction models in routine surveillance, and multiple regional, national, and international collaborations are needed.

PROBAST is a risk-of-bias assessment tool designed for systematically reviewing diagnostic or prognostic prediction models, and therefore, does not always fit well with infectious disease transmission risk models. It is reflected in the participant, analytic domain, and reporting domains. Although the aNOS scale showed better results in evaluation, it is ultimately not designed for predictive modeling studies. As several modeling studies have been conducted in the field of infectious diseases, which mainly use routine surveillance data, a better fit and acknowledged quality appraisal tool for them is needed.

### Implications for public health practice

All 11 reviewed prediction models showed a high risk of bias, and evidence from independent external validation of these models is currently lacking. Therefore, no any models could be recommended for use in practice at this point. It is anticipated that in the future, compiling individual regional data from multiple countries with similar transmission risks and healthcare systems could facilitate a better understanding of the general ability and implementation of prediction models. These data could be used to validate and update the currently available prediction models.

When building a new prediction model, it is recommend building on literature and expert opinion to select predictors rather than selecting them in a purely data-driven manner [51]. This is especially true for datasets with limited sample sizes. However, the application of data mining techniques may provide a way to generate potential predictors that are objective and reproducible. Based on multiple models identified in this review, researchers were encouraged to consider incorporating the following candidate predictors to build prediction models: vectorial, environmental and climatic, migration, surveillance and response related factors. By pointing to the most important methodological challenges and issues in designing and reporting by the currently available models, this review has provided potentially useful summarization for further studies aiming to develop new and improved models or validate and update existing ones.

## Strengths and limitations

Although this is the first study to summarize the existing prediction models on malaria re-introduction risk in eliminated settings, some limitations need to be acknowledged. First, although this review included studies published in English and Chinese, an exclusion of study published in other language may potentially lead to the exclusion of models that could otherwise have been included in the review. Second, the strategy was broad and required the screening of a relatively large number of titles and abstracts. This review will be updated continuously to provide up-to-date information for healthcare decision-makers and professionals as more international research emerges over time. Moreover, the large heterogeneity in the methodology used for included prediction models did not allow a meta-analysis.

## Conclusion

Models predicting malaria re-introduction risk in eliminated settings identified in this review had similar predictors, including climatic and environmental, vectorial factors, population mobility, malaria surveillance and response capacity. Although population movement is well acknowledged as a risk factor associated with malaria re-introduction risk in eliminated settings, it is not frequently incorporated in the risk prediction models. The existing evidence on prediction models for malaria risk in eliminated settings is lacking generalizability due to the lack of external validation. Therefore, future emphasis should be placed on the external validation of the existing models.

## Supplementary Information

**Additional file 1: Appendix 1.** Search strategy and databases. **Appendix 2.** PROBAST: Assessment of Risk of Bias and Concerns Regarding Applicability. **Appendix 3.** Adapted version of the Newcastle-Ottawa Scalechecklist for assessing the quality of cross-sectional studies. **Appendix 4.** Exclude after reading the whole article. **Appendix 5.** Limitation of included studies.**Appendix 6.** Quality assessment of included studies using the PROBAST scale for predictive modeling studies. **Appendix 7.** The aNOS for assessing the quality of cross-sectional studies.

## Data Availability

All data generated or analysed during this study are included in this published article and its Additional file 1.
