# Supplementary material for: Predicting the risk of malaria re-introduction in countries certified malaria-free: a systematic review
Source: Malar J. 2023 Jun 6;22:175. doi: 10.1186/s12936-023-04604-4 (PMC10243267; doi:10.1186/s12936-023-04604-4)
Supplement: Supplementary file 1 — Additional file 1: Appendix 1. Search strategy and databases. Appendix 2. PROBAST: Assessment of Risk of Bias and Concerns Regarding Applicability. Appendix 3. Adapted version of the Newcastle-Ottawa Scalechecklist for assessing the quality of cross-sectional studies. Appendix 4. Exclude after reading the whole article. Appendix 5. Limitation of included studies.Appendix 6. Quality assessment of included studies using the PROBAST scale for predictive modeling studies. Appendix 7. The aNOS for assessing the quality of cross-sectional studies. [file 12936_2023_4604_MOESM1_ESM.docx]

**Predicting the risk of malaria in low transmission settings: a systematic review**

*Supplementary material*

**Contents**

[Appendix 1 1](#_Toc3223)

[Search strategy and databases 1](#_Toc5701)

[Appendix 2 2](#_Toc11065)

[PROBAST: Assessment of Risk of Bias and Concerns Regarding Applicability 4](#_Toc13337)

[Appendix 3 4](#_Toc26931)

[Adapted version of the Newcastle-Ottawa Scale (aNOS) checklist 4](#_Toc9705)

[Appendix 4 6](#_Toc14887)

[Exclude after reading the whole article 6](#_Toc17145)

[Appendix 5 2](#_Toc10705)7

[Limitations of the included studies 2](#_Toc15249)7

[Appendix 6 2](#_Toc10705)8

[Quality assessment of included studies using the PROBAST scale for predictive modeling studies 2](#_Toc15249)8

[Appendix 7 30](#_Toc5348)

[The aNOS for assessing the quality of cross sectional studies 30](#_Toc30948)

Appendix 1

## **Search strategy and databases**

| **Database** | **Search terms** | **No. of studies generated** |
| --- | --- | --- |
| Web of science | #1 TS=(Malaria OR Malaria, Vivax OR Malaria, Falciparum OR acute malaria)  #2 TS=(Prediction OR Predictive model OR Prediction model OR Risk prediction OR Risk score OR Risk calculation OR Risk assessment)  #3 #1AND#2 | 6180 |
| PubMed | #1 (((("Malaria"[Mesh]) OR ("Malaria, Vivax"[Mesh])) OR ("Malaria, Falciparum"[Mesh])) OR ("Acute malaria" [Supplementary Concept])) OR (malaria[Title/Abstract])  #2 ((((((Prediction[Title/Abstract]) OR (Prediction model[Title/Abstract])) OR (Predictive model[Title/Abstract])) OR (Risk prediction[Title/Abstract])) OR (Risk score[Title/Abstract])) OR (Risk calculation[Title/Abstract])) OR (Risk assessment)  #3 #1 AND #2 | 2159 |
| Cochrane | #1 (Malaria OR Malaria, Vivax OR Malaria, Falciparum OR Acute malaria OR malaria):kw  #2 (Prediction OR Predictive model OR Prediction model OR Risk prediction OR Risk score OR Risk calculation OR Risk assessment):kw  #3 #1 AND #2 | 176 |
| China National Knowledge Infrastructure Database (CNKI) | #1 Topics: malaria + Malaria, Vivax + Malaria, Falciparum  #2 Topics: 'forecasting*' + risk forecasting + risk scoring + risk calculation+ risk assessment + forecasting models  #3 #1AND#2 | 311 |
| Total | - | 8826 |

# Appendix 2

**PROBAST: Assessment of Risk of Bias and Concerns Regarding Applicability***

| **1. Participants** | **2. Predictors** | **3. Outcome** | **4. Analysis** |
| --- | --- | --- | --- |
| **Signaling questions** |  |  |  |
| 1.1.Were appropriate data sources used, e.g., cohort, RCT, or nested case–control study data? | 2.1.Were predictors defined and assessed in a similar way for all participants? | 3.1.Was the outcome determined appropriately? | 4.1.Were there a reasonable number of participants with the outcome? |
| 1.2.Were all inclusions and  exclusions of participants  appropriate? | 2.2.Were predictor assessments made without knowledge of outcome data? | 3.2.Was a prespecified or standard outcome definition used? | 4.2.Were continuous and categorical predictors handled appropriately? |
| - | 2.3. Are all predictors available at the time the model is intended to be used? | 3.3.Were predictors excluded from the outcome definition? | 4.3.Were all enrolled participants included in the analysis? |
| - | - | 3.4.Was the outcome defined and determined in a similar way for all participants? | 4.4.Were participants with missing data handled appropriately? |
| - | - | 3.5.Was the outcome determined without knowledge of predictor information? | 4.5.Was selection of predictors based on univariable analysis avoided?† |
| - | - | 3.6.Was the time interval between predictor assessment and outcome  determination appropriate? | 4.6.Were complexities in the data (e.g., censoring, competing risks, sampling of control participants) accounted for appropriately? |
| - | - | - | 4.7.Were relevant model performance measures evaluated appropriately? |
| - | - | - | 4.8.Were model overfitting, underfitting, and optimism in model performance accounted  for?† |
| - | - | - | 4.9.Do predictors and their assigned weights in the final model correspond to the results from the reported multivariable analysis?† |
| **ROB** |  |  |  |
| Selection of participants | Predictors or their assessment | Outcome or its determination | Analysis |
| **Applicability** |  |  |  |
| Included participants or setting does not match the review question | Definition, assessment, or timing of predictors does not match the review question | Its definition, timing, or determination does not match the review question | - |

RCT = randomized controlled trial; ROB = risk of bias.

* For further details, please see the explanation and elaboration document (27), available at Annals.org, and www.probast.org. Signaling questions are answered as yes, probably yes, probably no, no, or no information. ROB and concerns for applicability are rated as low, high, or unclear.

† Development studies only

**References**

Wolff RF, Moons KGM, Riley RD, et al. PROBAST: A Tool to Assess the Risk of Bias and Applicability of Prediction Model Studies. Ann Intern Med 2019;170(1):51-58.

# Appendix 3

**Adapted version of the Newcastle-Ottawa Scale (aNOS) checklist for assessing the quality of cross sectional studies**

*Note: A study can be awarded a maximum of one/two stars for each numbered item within the Selection and Outcome categories.*

**Selection: (Maximum 5 stars)**

1) Representativeness of the sample:

a) Truly representative of the average in the target population. * (all subjects or random sampling)

b) Somewhat representative of the average in the target population. * (nonrandom sampling)

c) Selected group of users.

d) No description of the sampling strategy.

2) Sample size:

a) Justified and satisfactory. *

b) Not justified.

3) Non-respondents:

a) Comparability between respondents and non-respondents characteristics is established, and the response rate is satisfactory. *

b) The response rate is unsatisfactory, or the comparability between respondents and non-respondents is unsatisfactory.

c) No description of the response rate or the characteristics of the responders and the non-responders.

4) Ascertainment of the exposure (risk factor):

a) Validated measurement tool. **

b) Non-validated measurement tool, but the tool is available or described.*

c) No description of the measurement tool.

**Comparability: (Maximum 2 stars)**

1) The subjects in different outcome groups are comparable, based on the study design or analysis. Confounding factors are controlled.

a) The study controls for the most important factor (select one). *

b) The study control for any additional factor. *

**Outcome: (Maximum 3 stars)**

1) Assessment of the outcome:

a) Independent blind assessment. **

b) Record linkage. **

c) Self report. *

d) No description.

2) Statistical test:

a) The statistical test used to analyze the data is clearly described and appropriate, and the measurement of the association is presented, including confidence intervals and the probability level (p value). *

b) The statistical test is not appropriate, not described or incomplete.

**References**

MODESTI P A, REBOLDI G, CAPPUCCIO F P, et al. Panethnic Differences in Blood Pressure in Europe: A Systematic Review and Meta-Analysis [J]. PLoS One, 2016, 11(1): e0147601.

# Appendix 4

## **Exclude after reading the whole article^[1-310]^:**

**1.** Study setting was not in malaria pre-elimination, elimination or prevention of re-introduction**^[1-227]^:**

[1] RIBEIRO J M, ROSSIGNOL P A, SPIELMAN A. Aedes aegypti: model for blood finding strategy and prediction of parasite manipulation [J]. Exp Parasitol, 1985, 60(1): 118-132.

[2] FANG Y, DENG D, JIANG B Q, et al. Gray modeling and gray prediction of malaria epidemic trends in Xuzhou region [J]. Chinese Journal of Parasitology and Parasitic Diseases, 1988.

[3] CHEN W J, SI Y Z, ZHONG H S. Application of a grey system GM(1,1) model to predict the incidence of malaria [J]. Hainan Medical Journal, 1990.

[4] FANG Y, DENG D, GU Z C, et al. Interval classification, prediction and modeling of decline stage of malaria incidence in Xuzhou area [J]. Chinese Journal of Parasitology and Parasitic Diseases, 1991.

[5] TAN Q, WEI M. Grey dynamic model of malaria prevalence and trend prediction in Baise City [J]. Modern Preventive Medicine, 1992.

[6] GU W D. [The residual correcting prediction of grey model for malaria cases in China] [J]. Chinese journal of parasitology & parasitic diseases, 1993, 11(4): 248-250.

[7] JIANG R F, YE Y D. GM(1,1) model predictive analysis of foreign disease information with incomplete data [J]. Chinese Journal of Frontier Health and Quarantine, 1994.

[8] ZHANG H X. Application of grey system prediction model for predicting areas of declining malaria incidence [J]. Journal of Public Health and Preventive Medicine, 1994, (2): 12-13.

[9] GATTON M, HOGARTH W, SAUL A, et al. A model for predicting the transmission rate of malaria from serological data [J]. J Math Biol, 1996, 34(8): 878-888.

[10] XU G J, KANG Y. Prediction of recent malaria epidemic trends in Sichuan Province using GM(1,1) model [J]. Parasitoses and Infectious Diseases, 1996, (3): 140.

[11] BECK L R, RODRIGUEZ M H, DISTER S W, et al. Assessment of a remote sensing-based model for predicting malaria transmission risk in villages of Chiapas, Mexico [J]. Am J Trop Med Hyg, 1997, 56(1): 99-106.

[12] TORRES-SORANDO L, RODRGUEZ D J J E M. Models of spatio-temporal dynamics in malaria [J]. 1997, 104(2-3): 231-240.

[13] NAKAZAWA M, OHMAE H, ISHII A, et al. Malaria infection and human behavioral factors: A stochastic model analysis for direct observation data in the Solomon Islands [J]. Am J Hum Biol, 1998, 10(6): 781-789.

[14] SNOW R W, GOUWS E, OMUMBO J, et al. Models to predict the intensity of Plasmodium falciparum transmission: applications to the burden of disease in Kenya [J]. Trans R Soc Trop Med Hyg, 1998, 92(6): 601-606.

[15] WANG W M, JIN X L, ZHANG X P, et al. Application of gray model in predicting the number of incidences of inter-day malaria [J]. Chinese Journal of Health Statistics, 1998, (4): 66.

[16] CRAIG M H, SNOW R W, LE SUEUR D. A climate-based distribution model of malaria transmission in sub-Saharan Africa [J]. Parasitol Today, 1999, 15(3): 105-111.

[17] DE VRIES P, NEV. The local Eco-Epidemiological Malaria Risk Assessment (Lemra) model; proceedings of the PROCEEDINGS OF THE SECTION EXPERIMENTAL AND APPLIED ENTOMOLOGY OF THE NETHERLANDS ENTOMOLOGICAL SOCIETY(NEV), VOL 11, 1999 [C].

[18] MUHE L, OLJIRA B, DEGEFU H, et al. Clinical algorithm for malaria during low and high transmission seasons [J]. Arch Dis Child, 1999, 81(3): 216-220.

[19] WANG W M, JIN X L, ZHANG X P, et al. Application of two statistical models in predicting the outbreak of Plasmodium inter vivax [J]. Jiangsu Journal of Preventive Medicine, 1999, (4): 15-17.

[20] YU Y Y, GAO Y, XIE F Q. Application of gray model GM(1,1,0.2) in medical statistical prediction [J]. Chinese Journal of Hospital Statistics, 2000, (3): 150-151.

[21] KLEINSCHMIDT I, SHARP B L, CLARKE G P Y, et al. Use of generalized linear mixed models in the spatial analysis of small-area malaria incidence rates in KwaZulu Natal, South Africa [J]. Am J Epidemiol, 2001, 153(12): 1213-1221.

[22] WANG W M, JIN X L, ZHANG X P, et al. Application of the trend seasonal model prediction method to the number of incidences of inter-day malaria [J]. Chinese Journal of Zoonoses, 2001, (2): 112-119.

[23] GAO C Y. A preliminary study on the current situation of malaria epidemic in China and a neural network model of the influence of meteorological factors on malaria incidence [D]; Third Military Medical University, 2002.

[24] YANG G, ZHOU X, MALONE J B, et al. GIS prediction model of malaria transmission in Jiangsu province [J]. Chinese journal of preventive medicine], 2002, 36(2): 103-105.

[25] YANG G J, ZHOU X N, J B M, J.C.MCCARROLL,, et al. A study on the prediction model of malaria prevalence GIS in Jiangsu Province [J]. Chinese Journal of Preventive Medicine, 2002.

[26] YANG G J, ZHOU X N, J B M, J.C.MCCARROLL,, et al. Multi-factor spatial composite model predicts the distribution trend of malaria endemic areas in China [J]. Chinese Journal of Parasitology and Parasitic Diseases, 2002.

[27] GAO C Y, XIONG H Y, YI D, et al. A preliminary study of intelligent neural network models for the influence of meteorological factors on the development of malaria [J]. Chinese Journal of Epidemiology, 2003.

[28] HASSAN A N, KENAWY M A, KAMAL H, et al. GIS-based prediction of malaria risk in Egypt [J]. Eastern Mediterranean health journal 2003, 9(4): 548-558.

[29] QIU J L, ZHENG J N, ZHAO Y W. Application of gray models to study the prevalence of malaria in China [J]. Chinese Journal of Frontier Health and Quarantine, 2003.

[30] ABEKU T A, DE VLAS S J, BORSBOOM G, et al. Effects of meteorological factors on epidemic malaria in Ethiopia: a statistical modelling approach based on theoretical reasoning [J]. Parasitology, 2004, 128: 585-593.

[31] QIU J L, ZHENG J N, ZHAO Y W. The Morbidity Analysis and Trend Prediction of Malaria in China Using the Grey Model Method [J]. Chinese Journal of Vector Biology and Control, 2004.

[32] TEKLEHAIMANOT H D, SCHWARTZ J, TEKLEHAIMANOT A, et al. Weather-based prediction of Plasmodium falciparum malaria in epidemic-prone regions of Ethiopia II. Weather-based prediction systems perform comparably to early detection systems in identifying times for interventions [J]. Malar J, 2004, 3.

[33] WEN L. Research on prediction of malaria epidemic and construction of a GIS-based malaria surveillance and early warning system in Hainan Province ,China [D]; Fourth Military Medical University, 2004.

[34] WEN L, XU D Z, LIN M H, et al. Prediction of malaria incidence in malaria epidemic area with time series models [J]. Journal of Air Force Medical University, 2004.

[35] XU X H, JIN X L. Application of GM(1,1) mathematical model in malaria outbreak prediction [J]. Journal of Pathogen Biology, 2005, (3): 178-179.

[36] XU X H, JIN X L, LI J L. Application of autoregressive mathematical models in predicting malaria outbreaks [J]. Journal of Mathematical Medicine, 2005.

[37] YAZOUME Y, MOSHE H, RAINER S. Malaria risk prediction in holoendemic area: The potential of small scale mathematical modeling [MIM-YY-27867] [J]. Acta Trop, 2005, 95: S82-S83.

[38] GEMPERLI A, VOUNATSOU P, SOGOBA N, et al. Malaria mapping using transmission models: application to survey data from Mali [J]. Am J Epidemiol, 2006, 163(3): 289-297.

[39] GOSONIU L, VOUNATSOU P, SOGOBA N, et al. Bayesian modelling of geostatistical malaria risk data [J]. GEOSPATIAL HEALTH, 2006, 1(1): 127-139.

[40] KIANG R, ADIMI F, SOLKA V, et al. Meteorological, environmental remote sensing and neural network analysis of the epidemiology of malaria transmission in Thailand [J]. GEOSPATIAL HEALTH, 2006, 1(1): 71-84.

[41] KONCHOM S, SINGHASIVANON P, KAEWKUNGWAL J, et al. Early detection of malaria in an endemic area: model development [J]. The Southeast Asian journal of tropical medicine and public health, 2006, 37(6): 1067-1071.

[42] LI W J, YI X M, GU W W. Prediction of malaria epidemic trends in a city using a gray model [J]. Journal of Yangtze University(Natural Science Edition)Medicine V, 2006, (6): 282-283+285.

[43] QU J W, LIU J P, DU J, et al. Application of GM(1,1) grey model in malaria epidemic prediction [J]. Med Soc (Berkeley), 2006, (11): 5-7.

[44] ROSS A, MAIRE N, MOLINEAUX L, et al. An epidemiologic model of severe morbidity and mortality caused by Plasmodium falciparum [J]. Am J Trop Med Hyg, 2006, 75(2): 63-73.

[45] SMITH T, ROSS A, MAIRE N, et al. An epidemiologic model of the incidence of acute illness in Plasmodium falciparum malaria [J]. Am J Trop Med Hyg, 2006, 75(2): 56-62.

[46] WNAG W M, JIN X L, ZHOU H Y, et al. Application of Markov models in predicting malaria incidence trends [J]. China Tropical Medicine, 2006, (10): 1780-1781.

[47] XU X H, JIN X L. An autoregressive mathematical model for malaria outbreak prediction [J]. Chinese Journal of Health Statistics, 2006, (3): 228+239.

[48] GAO S T, LIU J P, ZHANG R L, et al. Establishment of GM(1,1) gray model for malaria epidemic prediction and analysis of its application effect [J]. Journal of Pathogen Biology, 2007, (5): 357-359.

[49] JURY M R, KANEMBA A D. A climate-based model for malaria prediction in southeastern Africa [J]. SOUTH AFRICAN JOURNAL OF SCIENCE, 2007, 103(1): 57-62.

[50] LI J L, GAO Q, JIN X L, et al. Analysis of prevalence of malaria in mobile population by grey prediction mathematical model [J]. China Tropical Medicine, 2007, (6): 872-873.

[51] MISHRA S K, PANIGRAHI P, MISHRA R, et al. Prediction of outcome in adults with severe falciparum malaria: a new scoring system [J]. Malar J, 2007, 6.

[52] ZHOU S S, HUANG F, SHEN Y Z. Application of ARIMA model in prediction of malaria incidence [J]. Journal of Pathogen Biology, 2007, (4): 284-286.

[53] ZHU J M, TANG L H, ZHOU S S, et al. Study on the Feasibility for ARIMA Model Application to Predict Malaria Incidence in an Unstable Malaria Area [J]. Chinese Journal of Parasitology and Parasitic Diseases, 2007, (3): 232-236.

[54] BRIET O J T, VOUNATSOU P, GUNAWARDENA D M, et al. Models for short term malaria prediction in Sri Lanka [J]. Malar J, 2008, 7.

[55] HAGHDOOST A A, ALEXANDER N, COX J. Modelling of malaria temporal variations in Iran [J]. Trop Med Int Health, 2008, 13(12): 1501-1508.

[56] HE F T, SHEN Y P. Three models predict future trends in malaria incidence in China [J]. Shanghai Journal of Preventive Medicine, 2008, (2): 57-60.

[57] NOOR A M, CLEMENTS A C A, GETHING P W, et al. Spatial prediction of Plasmodium falciparum prevalence in Somalia [J]. Malar J, 2008, 7.

[58] PERISSE A R S, STRICKLAND G T. Usefulness of clinical algorithm as screening process to detected malaria in low-to-moderate transmission areas of scarce health related resources [J]. Acta Trop, 2008, 107(3): 224-229.

[59] SILUE K D, RASO G, YAPI A, et al. Spatially-explicit risk profiling of Plasmodium falciparum infections at a small scale: a geostatistical modelling approach [J]. Malar J, 2008, 7.

[60] SMITH T, MAIRE N, ROSS A, et al. Towards a comprehensive simulation model of malaria epidemiology and control [J]. Parasitology, 2008, 135(13): 1507-1516.

[61] TIAN L W, BI Y, HO S C, et al. One-year delayed effect of fog on malaria transmission: a time-series analysis in the rain forest area of Mengla County, south-west China [J]. Malar J, 2008, 7.

[62] VEIGA N, GASPARETTO D. Spacial and temporal analysis model of malaria prevalence approach in Braganca and Augusto Correa - PA, between 2001 a 2006 [J]. SCRIPTA NOVA-REVISTA ELECTRONICA DE GEOGRAFIA Y CIENCIAS SOCIALES, 2008, 12(270).

[63] WEN L, SHI R H, FANG L Q, et al. Preliminary analysis of environmental influences on the spatial distribution of malaria prevalence in Hainan Province [J]. Chinese Journal of Epidemiology, 2008, (6): 581-585.

[64] CHATTERJEE C, SARKAR R R. Multi-Step Polynomial Regression Method to Model and Forecast Malaria Incidence [J]. PLoS One, 2009, 4(3).

[65] ELYAZAR I, ROGAYAH H, GETHING P, et al. SPATIAL PREDICTION OF PLASMODIUM FALCIPARUM PREVALENCE IN INDONESIA IN 2008 [J]. Am J Trop Med Hyg, 2009, 81(5): 52-52.

[66] GAUDART J, TOURE O, DESSAY N, et al. Modelling malaria incidence with environmental dependency in a locality of Sudanese savannah area, Mali [J]. Malar J, 2009, 8.

[67] GOSONIU L, VOUNATSOU P, SOGOBA N, et al. Mapping malaria risk in West Africa using a Bayesian nonparametric non-stationary model [J]. COMPUTATIONAL STATISTICS & DATA ANALYSIS, 2009, 53(9): 3358-3371.

[68] HE F T, SHEN Y P. Analysis of the trend of malaria in China from 1984 to 2007 and forecast of future incidence [J]. Modern Preventive Medicine, 2009, 36(23): 4407-4410+4413.

[69] JIANG Z H, LI J H. Application of GM(1,-1)-model in predicting malaria incidence in Guangxi [J]. Applied Preventive Medicine, 2009, 15(5): 305-307.

[70] MOHAPATRA M K, DAS S P. The malaria severity score: a method for severity assessment and risk prediction of hospital mortality for falciparum malaria in adults [J]. The Journal of the Association of Physicians of India, 2009, 57: 119-126.

[71] NOOR A M, GETHING P W, ALEGANA V A, et al. The risks of malaria infection in Kenya in 2009 [J]. BMC Infect Dis, 2009, 9.

[72] RASO G, SILUE K D, VOUNATSOU P, et al. Spatial risk profiling of Plasmodium falciparum parasitaemia in a high endemicity area in Cote d'Ivoire [J]. Malar J, 2009, 8.

[73] YE Y, HOSHEN M, KYOBUTUNGI C, et al. Local scale prediction of Plasmodium falciparum malaria transmission in an endemic region using temperature and rainfall [J]. GLOBAL HEALTH ACTION, 2009, 2: 103-115.

[74] ADIMI F, SOEBIYANTO R P, SAFI N, et al. Towards malaria risk prediction in Afghanistan using remote sensing [J]. 2010, 9(1): 1-11.

[75] DA CUNHA G B, LUITGARDS-MOURA J F, NAVES E L M, et al. Use of an artificial neural network to predict the incidence of malaria in the City of Canta, State of Roraima [J]. Rev Soc Bras Med Trop, 2010, 43(5): 567-570.

[76] GOSONIU L, VETA A M, VOUNATSOU P. Bayesian Geostatistical Modeling of Malaria Indicator Survey Data in Angola [J]. PLoS One, 2010, 5(3).

[77] HAQUE U, MAGALHAES R J S, REID H L, et al. Spatial prediction of malaria prevalence in an endemic area of Bangladesh [J]. Malar J, 2010, 9.

[78] LOHA E, JOURNAL B L J M. Model variations in predicting incidence of Plasmodium falciparum malaria using 1998-2007 morbidity and meteorological data from south Ethiopia [J]. 2010, 9(1): 166-166.

[79] LOHA E, LINDTJøRN B. Model variations in predicting incidence of Plasmodium falciparum malaria using 1998-2007 morbidity and meteorological data from south Ethiopia [J]. Malar J, 2010, 9: 166.

[80] NGOM R, SIEGMUND A. Urban malaria in Africa: an environmental and socio-economic modelling approach for Yaounde, Cameroon [J]. NATURAL HAZARDS, 2010, 55(3): 599-619.

[81] REID H, HAQUE U, CLEMENTS A C A, et al. Mapping Malaria Risk in Bangladesh Using Bayesian Geostatistical Models [J]. Am J Trop Med Hyg, 2010, 83(4): 861-867.

[82] RIEDEL N, VOUNATSOU P, MILLER J M, et al. Geographical patterns and predictors of malaria risk in Zambia: Bayesian geostatistical modelling of the 2006 Zambia national malaria indicator survey (ZMIS) [J]. Malar J, 2010, 9.

[83] SAFI N, ADIMI F, SOEBIYANTO R P, et al. TOWARD MALARIA RISK PREDICTION IN AFGHANISTAN USING REMOTE SENSING; proceedings of the NETWORKING THE WORLD WITH REMOTE SENSING, F 2010 [C].

[84] VELASQUEZ J O R. A method for forecasting the seasonal dynamic of malaria in the municipalities of Colombia [J]. REVISTA PANAMERICANA DE SALUD PUBLICA-PAN AMERICAN JOURNAL OF PUBLIC HEALTH, 2010, 27(3): 211-218.

[85] WANGDI K, SINGHASIVANON P, SILAWAN T, et al. Development of temporal modelling for forecasting and prediction of malaria infections using time-series and ARIMAX analyses: A case study in endemic districts of Bhutan [J]. Malar J, 2010, 9.

[86] YAMANA T K, ELTAHIR E A B. Early warnings of the potential for malaria transmission in rural Africa using the hydrology, entomology and malaria transmission simulator (HYDREMATS) [J]. Malar J, 2010, 9.

[87] ZHANG W Y. Predicting the transmission risk of hemorrhagic fever with renal syndrome and malaria based on environmental factors [D]; Chinese People's Liberation Army Academy of Military Medical Sciences, 2010.

[88] ACHCAR J A, MARTINEZ E Z, DE SOUZA A D P, et al. Use of Poisson spatiotemporal regression models for the Brazilian Amazon forest: malaria count data [J]. Rev Soc Bras Med Trop, 2011, 44(6): 749-754.

[89] CAO P G, NONG Z, JIANG Z H. Prediction of malaria incidence trend in Baise city by grey system theory GM(1,1) model [J]. China Tropical Medicine, 2011, 11(11): 1318-1319+1326.

[90] GOSONIU L, VOUNATSOU P. Non-stationary partition modeling of geostatistical data for malaria risk mapping [J]. JOURNAL OF APPLIED STATISTICS, 2011, 38(1): 3-13.

[91] GUO H Q, YU H L, QU B, et al. A grey prediction model study of national malaria incidence from 1988-2010 [J]. China Tropical Medicine, 2011, 11(6): 639-640+643.

[92] LIU J, QU B, HE Q C. Analysis and prediction of malaria epidemic in China from 2004 to 2009 by applying time series model [J]. Chinese Journal of Vector Biology and Control, 2011, 22(2): 134-136+140.

[93] MATEUS J C, CARRASQUILLA G. Predictors of local malaria outbreaks: an approach to the development of an early warning system in Colombia [J]. Mem Inst Oswaldo Cruz, 2011, 106 Suppl 1(Suppl 1): 107-113.

[94] STENSGAARD A S, VOUNATSOU P, ONAPA A W, et al. Bayesian geostatistical modelling of malaria and lymphatic filariasis infections in Uganda: predictors of risk and geographical patterns of co-endemicity [J]. Malar J, 2011, 10.

[95] XIAO D. Spatio-temporal distribution, the construction and evaluation of prediction model on malaria epidemics in Hainan, China [D]; People's Liberation Army Air Force Military Medical University, 2011.

[96] GAO H W. Prediction of malaria transmission risk at different spatial and temporal scales and study of environmental impact factors [D]; Chinese People's Liberation Army Academy of Military Medical Sciences, 2012.

[97] GIARDINA F, GOSONIU L, KONATE L, et al. Estimating the Burden of Malaria in Senegal: Bayesian Zero-Inflated Binomial Geostatistical Modeling of the MIS 2008 Data [J]. PLoS One, 2012, 7(3).

[98] GOSONIU L, MSENGWA A, LENGELER C, et al. Spatially Explicit Burden Estimates of Malaria in Tanzania: Bayesian Geostatistical Modeling of the Malaria Indicator Survey Data [J]. PLoS One, 2012, 7(5).

[99] MIDEKISA A, SENAY G, HENEBRY G M, et al. Remote sensing-based time series models for malaria early warning in the highlands of Ethiopia [J]. Malar J, 2012, 11.

[100] MUSA M I, SHOHAIMI S, HASHIM N R, et al. A climate distribution model of malaria transmission in Sudan [J]. GEOSPATIAL HEALTH, 2012, 7(1): 27-36.

[101] SHANG X P, HUAXUN Z, LI K J, et al. Fit analysis of trend seasonal model to predict local inter-day malaria cases in Hubei Province [J]. Journal of Pathogen Biology, 2012, 7(4): 274-275+286.

[102] ALEGANA V A, ATKINSON P M, WRIGHT J A, et al. Estimation of malaria incidence in northern Namibia in 2009 using Bayesian conditional-autoregressive spatial-temporal models [J]. Spat Spatiotemporal Epidemiol, 2013, 7: 25-36.

[103] BRIET O J T, AMERASINGHE P H, VOUNATSOU P. Generalized Seasonal Autoregressive Integrated Moving Average Models for Count Data with Application to Malaria Time Series with Low Case Numbers [J]. PLoS One, 2013, 8(6).

[104] ERMERT V, FINK A H, PAETH H. The potential effects of climate change on malaria transmission in Africa using bias-corrected regionalised climate projections and a simple malaria seasonality model [J]. CLIMATIC CHANGE, 2013, 120(4): 741-754.

[105] FAN W J. Application of mathematical model in the forecasting of malaria incidence and incidence trend analysis on malaria in Hefei [D]; AnHui Medical University, 2013.

[106] FAN W J, LU Q, ZOU L W, et al. Application of Markov model in predicting the trend of malaria incidence in Hefei city [J]. China Tropical Medicine, 2013, 13(7): 819-821.

[107] FAN W J, LU Q, ZOU L W, et al. Application of ARIMA model in predicting malaria incidence in Hefei city [J]. Acta Universitatis Medicinalis Anhui, 2013, 48(3): 252-256.

[108] MOIROUX N, BIO-BANGANA A S, DJENONTIN A, et al. Modelling the risk of being bitten by malaria vectors in a vector control area in southern Benin, west Africa [J]. PARASITES & VECTORS, 2013, 6.

[109] RAI P K, NATHAWAT M S, RAI S. Using the information value method in a geographic information system and remote sensing for malaria mapping: a case study from India [J]. Inform Prim Care, 2013, 21(1): 43-52.

[110] RODRIGUEZ D J, DELGADO L, RAMOS S, et al. A model for the dynamics of malaria in Paria Peninsula, Sucre State, Venezuela [J]. Ecol Modell, 2013, 259: 1-9.

[111] ROSAS-AGUIRRE A, LLANOS-CUENTAS A, SPEYBROECK N, et al. Assessing malaria transmission in a low endemicity area of north-western Peru [J]. Malar J, 2013, 12.

[112] VILLALTA D, GUENNI L, RUBIO-PALIS Y, et al. Bayesian space-time modeling of malaria incidence in Sucre state, Venezuela SPATIAL SPECIAL ISSUE [J]. ASTA-ADVANCES IN STATISTICAL ANALYSIS, 2013, 97(2): 151-171.

[113] ZACARIAS O P, BOSTROM H, IEEE. Comparing Support Vector Regression and Random Forests for Predicting Malaria Incidence in Mozambique; proceedings of the 2013 INTERNATIONAL CONFERENCE ON ADVANCES IN ICT FOR EMERGING REGIONS (ICTER), F 2013, 2013 [C].

[114] GITHEKO A K, OGALLO L, LEMNGE M, et al. Development and validation of climate and ecosystem-based early malaria epidemic prediction models in East Africa [J]. Malar J, 2014, 13.

[115] KIENBERGER S, HAGENLOCHER M. Spatial-explicit modeling of social vulnerability to malaria in East Africa [J]. INTERNATIONAL JOURNAL OF HEALTH GEOGRAPHICS, 2014, 13.

[116] LAUDERDALE J M, CAMINADE C, HEATH A E, et al. Towards seasonal forecasting of malaria in India [J]. Malar J, 2014, 13.

[117] MOHAPATRA B N, JANGID S K, MOHANTY R. GCRBS score: a new scoring system for predicting outcome in severe falciparum malaria [J]. The Journal of the Association of Physicians of India, 2014, 62(1): 14-17.

[118] NYGREN D, STOYANOV C, LEWOLD C, et al. Remotely-sensed, nocturnal, dew point correlates with malaria transmission in Southern Province, Zambia: a time-series study [J]. Malar J, 2014, 13.

[119] RAO M S, MURTY U S, RAO K M, et al. Assessment of malaria incidence using the Richards model in Arunachal Pradesh, India [J]. Epidemiol Infect, 2014, 142(10): 2227-2236.

[120] RUMISHA S F, SMITH T, ABDULLA S, et al. Modelling heterogeneity in malaria transmission using large sparse spatio-temporal entomological data [J]. GLOBAL HEALTH ACTION, 2014, 7.

[121] VALLE D, LIMA J M T. Large-scale drivers of malaria and priority areas for prevention and control in the Brazilian Amazon region using a novel multi-pathogen geospatial model [J]. Malar J, 2014, 13.

[122] ZHOU L, ZHANG F, WANG Y, et al. A neural network model for the prediction of malaria in Jingmen City, China [J]. Int J Infect Dis, 2014, 21: 270-270.

[123] ADIGUN A B, GAJERE E N, ORESANYA O, et al. Malaria risk in Nigeria: Bayesian geostatistical modelling of 2010 malaria indicator survey data [J]. Malar J, 2015, 14.

[124] BHAVNANI D, TREJO B, PEREZ L M, et al. PREDICTIVE MALARIA RISK MODELING USING AGGREGATE CASE DATA FOR IMPROVED INTERVENTION TARGETING IN HONDURAS [J]. Am J Trop Med Hyg, 2015, 93(4): 470-470.

[125] CHIROMA H, ABDUL-KAREEM S, IBRAHIM U, et al. MALARIA SEVERITY CLASSIFICATION THROUGH JORDAN-ELMAN NEURAL NETWORK BASED ON FEATURES EXTRACTED FROM THICK BLOOD SMEAR [J]. NEURAL NETWORK WORLD, 2015, 25(5): 565-584.

[126] GIARDINA F, FRANKE J, VOUNATSOU P. Geostatistical modelling of the malaria risk in Mozambique: effect of the spatial resolution when using remotely-sensed imagery [J]. GEOSPATIAL HEALTH, 2015, 10(2): 232-238.

[127] MACERA M A C, LOUZADA F, CANCHO V G, et al. The exponential-Poisson model for recurrent event data: An application to a set of data on malaria in Brazil [J]. Biom J, 2015, 57(2): 201-214.

[128] ONYIRI N. Estimating malaria burden in Nigeria: a geostatistical modelling approach [J]. GEOSPATIAL HEALTH, 2015, 10(2): 163-170.

[129] PINCHOFF J, CHAPONDA M, SHIELDS T, et al. Predictive Malaria Risk and Uncertainty Mapping in Nchelenge District, Zambia: Evidence of Widespread, Persistent Risk and Implications for Targeted Interventions [J]. Am J Trop Med Hyg, 2015, 93(6): 1260-1267.

[130] ROY M, BOUMA M, DHIMAN R C, et al. Predictability of epidemic malaria under non-stationary conditions with process-based models combining epidemiological updates and climate variability [J]. Malar J, 2015, 14.

[131] SONG Y Z. Detecting predictors and future risk prediction for malaria incidences in Northern China using remote sensing data [D]; China University of Geosciences (Beijing), 2015.

[132] XIA J. Research on spatio-temporal distribution and prediction for malaria epidemics in Hubei Province, China [D]; Huazhong University of Science and Technology 2015.

[133] ZINSZER K, KIGOZI R, CHARLAND K, et al. Forecasting malaria in a highly endemic country using environmental and clinical predictors [J]. Malar J, 2015, 14.

[134] ANWAR M Y, LEWNARD J A, PARIKH S, et al. Time series analysis of malaria in Afghanistan: using ARIMA models to predict future trends in incidence [J]. Malar J, 2016, 15.

[135] ASARE E O, TOMPKINS A M, AMEKUDZI L K, et al. A breeding site model for regional, dynamical malaria simulations evaluated using in situ temporary ponds observations [J]. GEOSPATIAL HEALTH, 2016, 11: 56-66.

[136] GOMES E, CAPINHA C, ROCHA J, et al. Mapping Risk of Malaria Transmission in Mainland Portugal Using a Mathematical Modelling Approach [J]. PLoS One, 2016, 11(11).

[137] HADDAWY P, KASANTIKUL R, HASAN A H M I, et al. Spatiotemporal Bayesian Networks for Malaria Prediction: Case Study of Northern Thailand [J]. Stud Health Technol Inform, 2016, 228: 773-777.

[138] HAMMER, TAMAS, TRAJER, et al. Climate-based seasonality model of temperate malaria based on the epidemiological data of 1927-1934, Hungary [J]. 2016.

[139] HASAN A, HADDAWY P, KAMINKA G A, et al. Integrating ARIMA and Spatiotemporal Bayesian Networks for High Resolution Malaria Prediction; proceedings of the ECAI 2016: 22ND EUROPEAN CONFERENCE ON ARTIFICIAL INTELLIGENCE, F 2016, 2016 [C].

[140] HOUNGBEDJI C A, CHAMMARTIN F, YAPI R B, et al. Spatial mapping and prediction of Plasmodium falciparum infection risk among school-aged children in Cote d'Ivoire [J]. PARASITES & VECTORS, 2016, 9.

[141] JING X, HUA-XUN Z, WEN L, et al. Application of ARIMA model on prediction of malaria incidence [J]. Chinese journal of schistosomiasis control, 2016, 28(2): 135-140.

[142] KAPWATA T, GEBRESLASIE M T. Random forest variable selection in spatial malaria transmission modelling in Mpumalanga Province, South Africa [J]. GEOSPATIAL HEALTH, 2016, 11(3): 251-262.

[143] KARURI S W, SNOW R W. Forecasting paediatric malaria admissions on the Kenya Coast using rainfall [J]. GLOBAL HEALTH ACTION, 2016, 9.

[144] MACHERERA M, CHIMBARI M J. Developing a community-centred malaria early warning system based on indigenous knowledge: Gwanda District, Zimbabwe [J]. Jamba (Potchefstroom, South Africa), 2016, 8(1): 289.

[145] OKAMI S, KOHTAKE N. Fine-Scale Mapping by Spatial Risk Distribution Modeling for Regional Malaria Endemicity and Its Implications under the Low-to-Moderate Transmission Setting in Western Cambodia [J]. PLoS One, 2016, 11(7).

[146] OSTOVAR A, HAGHDOOST A A, RAHIMIFOROUSHANI A, et al. Time Series Analysis of Meteorological Factors Influencing Malaria in South Eastern Iran [J]. JOURNAL OF ARTHROPOD-BORNE DISEASES, 2016, 10(2): 222-237.

[147] SANTOS-VEGA M, BOUMA M J, KOHLI V, et al. Population Density, Climate Variables and Poverty Synergistically Structure Spatial Risk in Urban Malaria in India [J]. PLoS Negl Trop Dis, 2016, 10(12).

[148] SEMAKULA H M, SONG G B, ACHUU S P, et al. A Bayesian belief network modelling of household factors influencing the risk of malaria: A study of parasitaemia in children under five years of age in sub-Saharan Africa [J]. ENVIRONMENTAL MODELLING & SOFTWARE, 2016, 75: 59-67.

[149] TOMPKINS A M, CAPORASO L. Assessment of malaria transmission changes in Africa, due to the climate impact of land use change using Coupled Model Intercomparison Project Phase 5 earth system models [J]. GEOSPATIAL HEALTH, 2016, 11: 6-17.

[150] VITOR-SILVA S, SIQUEIRA A M, SAMPAIO V D, et al. Declining malaria transmission in rural Amazon: changing epidemiology and challenges to achieve elimination [J]. Malar J, 2016, 15.

[151] XIA J, ZHANG H X, LIN W, et al. Application of ARIMA model on prediction of malaria incidence [J]. Chinese Journal of Schistosomiasis Control, 2016, 28(2): 135-140.

[152] DARKOH E L, LARBI J A, LAWER E A. A Weather-Based Prediction Model of Malaria Prevalence in Amenfi West District, Ghana [J]. Malar Res Treat, 2017, 2017: 7820454.

[153] FERRAO J L, MENDES J M, PAINHO M. Modelling the influence of climate on malaria occurrence in Chimoio Municipality, Mozambique [J]. PARASITES & VECTORS, 2017, 10.

[154] HUI-YU H, HUA-QIN S, SHUN-XIAN Z, et al. Application of ARIMA model to predict number of malaria cases in China [J]. Chinese journal of schistosomiasis control, 2017, 29(4): 436-440.

[155] KUMAR R, DASH C, RANI K. Ecological covariates based predictive model of malaria risk in the state of Chhattisgarh, India [J]. Journal of parasitic diseases : official organ of the Indian Society for Parasitology, 2017, 41(3): 761-767.

[156] KURSAH M B. Modelling malaria susceptibility using geographic information system [J]. GEOJOURNAL, 2017, 82(6): 1101-1111.

[157] LAGUNA F, GRILLET M E, LEON J R, et al. Modelling malaria incidence by an autoregressive distributed lag model with spatial component [J]. SPATIAL AND SPATIO-TEMPORAL EPIDEMIOLOGY, 2017, 22: 27-37.

[158] RODRíGUEZ-VELáSQUEZ J O, PRIETO-BOHóRQUEZ S E, CORREA-HERRERA S C, et al. Dynamics of the malaria epidemic in Colombia: temporal probabilistic prediction [J]. Revista de salud publica (Bogota, Colombia), 2017, 19(1): 52-59.

[159] SEMAKULA H M, SONG G B, ACHUU S P, et al. Prediction of future malaria hotspots under climate change in sub-Saharan Africa [J]. CLIMATIC CHANGE, 2017, 143(3): 415-428.

[160] SEWE M O, TOZAN Y, AHLM C, et al. Using remote sensing environmental data to forecast malaria incidence at a rural district hospital in Western Kenya [J]. Sci Rep, 2017, 7.

[161] SMITH J, TAHANI L, BOBOGARE A, et al. Malaria early warning tool: linking inter-annual climate and malaria variability in northern Guadalcanal, Solomon Islands [J]. Malar J, 2017, 16.

[162] VALLE D, AMRATIA P, MILLAR J, et al. NOVEL MODELING APPROACHES TO IMPROVE SPATIAL PREDICTIONS OF MALARIA PREVALENCE [J]. Am J Trop Med Hyg, 2017, 97(5): 521-521.

[163] WEN L, LIN M H, LI C Y, et al. Effectiveness of back propagation neural network model and stepwise regression in prediction of malaria incidence with meteorological factors [J]. Chinese Journal of Public Health, 2017, 33(6): 942-945.

[164] HADDAWY P, HASAN A, KASANTIKUL R, et al. Spatiotemporal Bayesian networks for malaria prediction [J]. Artif Intell Med, 2018, 84: 127-138.

[165] HADDAWY P, YIN M S, WISANRAKKIT T, et al. Complexity-Based Spatial Hierarchical Clustering for Malaria Prediction [J]. Journal of healthcare informatics research, 2018, 2(4): 423-447.

[166] JEANNE I, CHAMBERS L E, KAZAZIC A, et al. Mapping a Plasmodium transmission spatial suitability index in Solomon Islands: a malaria monitoring and control tool [J]. Malar J, 2018, 17.

[167] MACHARIA P M, GIORGI E, NOOR A M, et al. Spatio-temporal analysis of Plasmodium falciparum prevalence to understand the past and chart the future of malaria control in Kenya [J]. Malar J, 2018, 17.

[168] MILLAR J, PSYCHAS P, ABUAKU B, et al. Detecting local risk factors for residual malaria in northern Ghana using Bayesian model averaging [J]. Malar J, 2018, 17.

[169] VERMA M, KISHORE K, KUMAR M, et al. Google Search Trends Predicting Disease Outbreaks: An Analysis from India [J]. HEALTHCARE INFORMATICS RESEARCH, 2018, 24(4): 300-+.

[170] ZHAI J X, LU Q, HU W B, et al. Development of an empirical model to predict malaria outbreaks based on monthly case reports and climate variables in Hefei, China, 1990-2011 [J]. Acta Trop, 2018, 178: 148-154.

[171] ZHENG J X. Modelling the malaria transmission and risk prediction of malaria in Yunnan border area [D]; Jiangsu Institute of Parasitic Diseases 2018.

[172] ADEOLA A M, BOTAI J O, OLWOCH J M, et al. Predicting malaria cases using remotely sensed environmental variables in Nkomazi, South Africa [J]. GEOSPATIAL HEALTH, 2019, 14(1): 81-91.

[173] FRASER M, LANKIA J L, BETANCOURT M, et al. USING PREDICTIVE MODELING FOR THE PROACTIVE IDENTIFICATION OF MALARIA HOTSPOTS IN SENEGAL [J]. Am J Trop Med Hyg, 2019, 101: 307-307.

[174] KIFLE M M, TEKLEMARIAM T T, TEWELDEBERHAN A M, et al. Malaria Risk Stratification and Modeling the Effect of Rainfall on Malaria Incidence in Eritrea [J]. J Environ Public Health, 2019, 2019.

[175] KIM Y, RATNAM J V, DOI T, et al. Malaria predictions based on seasonal climate forecasts in South Africa: A time series distributed lag nonlinear model [J]. Sci Rep, 2019, 9.

[176] LUCAS T C D, NANDI A, NGUYEN M, et al. USING MACHINE LEARNING PREDICTIONS OF MALARIA PREVALENCE TO IMPROVE GEOSTATISTICAL DISAGGREGATION MODELS OF INCIDENCE [J]. Trans R Soc Trop Med Hyg, 2019, 113: S62-S62.

[177] QUARTEY-PAPAFIO T K, LIU S, JAVED S J G S T, et al. Grey relational evaluation of impact and control of malaria in Sub-Saharan Africa [J]. 2019, 9(4): 415-431.

[178] SOLANO-VILLARREAL E, VALDIVIA W, PEARCY M, et al. Malaria risk assessment and mapping using satellite imagery and boosted regression trees in the Peruvian Amazon [J]. Sci Rep, 2019, 9.

[179] WANG M Y, WANG H, WANG J, et al. A novel model for malaria prediction based on ensemble algorithms [J]. PLoS One, 2019, 14(12).

[180] WANG X L, CAO J B, LI D D, et al. Management of imported malaria cases and healthcare institutions in central China, 2012-2017: application of decision tree analysis [J]. Malar J, 2019, 18(1).

[181] YE Y, LAO F X, WEI S L, et al. Epidemiological analysis and Markov model prediction of malaria epidemics from 1950 to 2014 in Heng County，Guangxi [J]. Progress in Microbiology and Immunology, 2019, 47(1): 54-59.

[182] ATEBA F F, FEBRERO-BANDE M, SAGARA I, et al. Predicting Malaria Transmission Dynamics in Dangassa, Mali: A Novel Approach Using Functional Generalized Additive Models [J]. Int J Environ Res Public Health, 2020, 17(17).

[183] BROWN B J, MANESCU P, PRZYBYLSKI A A, et al. Data-driven malaria prevalence prediction in large densely populated urban holoendemic sub-Saharan West Africa [J]. Sci Rep, 2020, 10(1).

[184] KIM Y, RATNAM J V, DOI T, et al. Malaria predictions based on seasonal climate forecasts in South Africa: A time series distributed lag nonlinear model (vol 9, 17882, 2019) [J]. Sci Rep, 2020, 10(1).

[185] KUMAR P, VATSA R, SARTHI P P, et al. Modeling an association between malaria cases and climate variables for Keonjhar district of Odisha, India: a Bayesian approach [J]. Journal of parasitic diseases : official organ of the Indian Society for Parasitology, 2020, 44(2): 319-331.

[186] KURNIANINGSIH, WIRASATRIYA A, LAZUARDI L, et al. IOD and ENSO-Related Time Series Variability and Forecasting of Dengue and Malaria Incidence in Indonesia; proceedings of the 2020 INTERNATIONAL SYMPOSIUM ON COMMUNITY-CENTRIC SYSTEMS (CCS), F 2020, 2020 [C].

[187] MASUD M, ALHUMYANI H, ALSHAMRANI S S, et al. Leveraging Deep Learning Techniques for Malaria Parasite Detection Using Mobile Application [J]. WIRELESS COMMUNICATIONS & MOBILE COMPUTING, 2020, 2020.

[188] MEENU M, SUBHASRI G, MAHIMA R. Machine Learning Based Malaria Prediction Using KNN Algorithm [J]. BIOSCIENCE BIOTECHNOLOGY RESEARCH COMMUNICATIONS, 2020, 13(2): 38-42.

[189] MORANG'A C M, AMENGA-ETEGO L, BAH S Y, et al. Machine learning approaches classify clinical malaria outcomes based on haematological parameters [J]. BMC Med, 2020, 18(1).

[190] NABET C, CHALINE A, FRANETICH J F, et al. Prediction of malaria transmission drivers in Anopheles mosquitoes using artificial intelligence coupled to MALDI-TOF mass spectrometry [J]. Sci Rep, 2020, 10(1).

[191] QUAN Q, WANG J X, LIU L L. An Effective Convolutional Neural Network for Classifying Red Blood Cells in Malaria Diseases [J]. INTERDISCIPLINARY SCIENCES-COMPUTATIONAL LIFE SCIENCES, 2020, 12(2): 217-225.

[192] ROUAMBA T, SAMADOULOUGOU S, KIRAKOYA-SAMADOULOUGOU F. Addressing challenges in routine health data reporting in Burkina Faso through Bayesian spatiotemporal prediction of weekly clinical malaria incidence [J]. Sci Rep, 2020, 10(1): 16568.

[193] SHI B Y, LIN S, TAN Q, et al. Inference and prediction of malaria transmission dynamics using time series data [J]. INFECTIOUS DISEASES OF POVERTY, 2020, 9(1).

[194] SLATER H C, FOY B D, KOBYLINSKI K, et al. Ivermectin as a novel complementary malaria control tool to reduce incidence and prevalence: a modelling study [J]. Lancet Infect Dis, 2020, 20(4): 498-508.

[195] SOW B, MUKHTAR H, AHMAD H F, et al. Assessing the relative importance of social determinants of health in malaria and anemia classi?cation based on machine learning techniques [J]. INFORMATICS FOR HEALTH & SOCIAL CARE, 2020, 45(3): 229-241.

[196] VERMA A K, KUPPILI V, SRIVASTAVA S K, et al. A new backpropagation neural network classification model for prediction of incidence of malaria [J]. FRONTIERS IN BIOSCIENCE-LANDMARK, 2020, 25: 299-334.

[197] AHETO J M K, DUAH H O, AGBADI P, et al. A predictive model, and predictors of under-five child malaria prevalence in Ghana: How do LASSO, Ridge and Elastic net regression approaches compare? [J]. PREVENTIVE MEDICINE REPORTS, 2021, 23.

[198] AITKEN E H, DAMELANG T, ORTEGA-PAJARES A, et al. Developing a multivariate prediction model of antibody features associated with protection of malaria-infected pregnant women from placental malaria [J]. ELIFE, 2021, 10.

[199] BBOSA F F, NABUKENYA J, NABENDE P, et al. On the goodness of fit of parametric and non-parametric data mining techniques: the case of malaria incidence thresholds in Uganda [J]. HEALTH AND TECHNOLOGY, 2021, 11(4): 929-940.

[200] CLEARY E, HETZEL M W, SIBA P, et al. Spatial prediction of malaria prevalence in Papua New Guinea: a comparison of Bayesian decision network and multivariate regression modelling approaches for improved accuracy in prevalence prediction [J]. Malar J, 2021, 20(1).

[201] DEELDER W, BENAVENTE E D, PHELAN J, et al. Using deep learning to identify recent positive selection in malaria parasite sequence data [J]. Malar J, 2021, 20(1).

[202] DONKOR E, KELLY M, ELIASON C, et al. A Bayesian Spatio-Temporal Analysis of Malaria in the Greater Accra Region of Ghana from 2015 to 2019 [J]. Int J Environ Res Public Health, 2021, 18(11).

[203] GONDWE T, YANG Y G, YOSEFE S, et al. Epidemiological Trends of Malaria in Five Years and under Children of Nsanje District in Malawi, 2015-2019 [J]. Int J Environ Res Public Health, 2021, 18(23).

[204] HARVEY D, VALKENBURG W, AMARA A. Predicting malaria epidemics in Burkina Faso with machine learning [J]. PLoS One, 2021, 16(6).

[205] IBRAHIM T B, EICHNER M, SCHNEIDER K A. SUSTAINING EFFICIENT MALARIA VECTOR CONTROL WHILE ACCOUNTING FOR BREEDING SITE STAGES: A PREDICTIVE MODELLING APPROACH [J]. Am J Trop Med Hyg, 2021, 105(5): 182-182.

[206] KIMUYU J S. Comparative spatial-temporal analysis and predictive modeling of climate change-induced malaria vectors' invasion in new hotspots in Kenya [J]. SN APPLIED SCIENCES, 2021, 3(8).

[207] LEE Y W, CHOI J W, SHIN E H. Machine learning model for predicting malaria using clinical information [J]. Comput Biol Med, 2021, 129.

[208] LIMA M V M, LAPORTA G Z. Evaluation of prediction models for the occurrence of malaria in the state of Amapá, Brazil, 1997-2016: an ecological study [J]. Epidemiol Serv Saude, 2021, 30(1): e2020080.

[209] MCLAUGHLIN M, PELLé K G, SCARPINO S V, et al. Development and Validation of Manually Modified and Supervised Machine Learning Clinical Assessment Algorithms for Malaria in Nigerian Children [J]. Frontiers in artificial intelligence, 2021, 4: 554017.

[210] OKAGBUE H I, OGUNTUNDE P E, OBASI E C M, et al. Diagnosing malaria from some symptoms: a machine learning approach and public health implications [J]. HEALTH AND TECHNOLOGY, 2021, 11(1): 23-37.

[211] REIKER T, GOLUMBEANU M, SHATTOCK A, et al. Emulator-based Bayesian optimization for efficient multi-objective calibration of an individual-based model of malaria [J]. NATURE COMMUNICATIONS, 2021, 12(1).

[212] TANEJA S B, DOUGLAS G P, COOPER G F, et al. Bayesian network models with decision tree analysis for management of childhood malaria in Malawi [J]. BMC Med Inform Decis Mak, 2021, 21(1).

[213] TOHIDINIK H R, KESHAVARZ H, MOHEBALI M, et al. Prediction of malaria cases in the southeastern Iran using climatic variables: An 18-year SARIMA time series analysis [J]. Asian Pac J Trop Med, 2021, 14(10): 463-470.

[214] YADAV S S, KADAM V J, JADHAV S M, et al. Machine Learning based Malaria Prediction using Clinical Findings; proceedings of the 2021 INTERNATIONAL CONFERENCE ON EMERGING SMART COMPUTING AND INFORMATICS (ESCI), F 2021, 2021 [C].

[215] ENDO A, AMARASEKARE P. Predicting the Spread of Vector-Borne Diseases in a Warming World [J]. FRONTIERS IN ECOLOGY AND EVOLUTION, 2022, 10.

[216] FALL P, DIOUF I, DEME A, et al. Assessment of Climate-Driven Variations in Malaria Transmission in Senegal Using the VECTRI Model [J]. ATMOSPHERE, 2022, 13(3).

[217] JIMOH R G, ABISOYE O A, UTHMAN M M B. Ensemble Feed-Forward Neural Network and Support Vector Machine for Prediction of Multiclass Malaria Infection [J]. JOURNAL OF INFORMATION AND COMMUNICATION TECHNOLOGY-MALAYSIA, 2022, 21(1): 117-148.

[218] LAI X Y, QIAN J. A study on prediction of malaria incidence based on a combined machine learning model [J]. Mathematics in Practice and Theory, 2022, 52(2): 125-133.

[219] MARIKI M, MKOBA E, MDUMA N. Combining Clinical Symptoms and Patient Features for Malaria Diagnosis: Machine Learning Approach [J]. APPLIED ARTIFICIAL INTELLIGENCE, 2022, 36(1).

[220] MOHAMED J, MOHAMED A I, DAUD E I. Evaluation of prediction models for the malaria incidence in Marodijeh Region, Somaliland [J]. Journal of parasitic diseases : official organ of the Indian Society for Parasitology, 2022, 46(2): 395-408.

[221] MOHAPATRA P, TRIPATHI N K, PAL I, et al. Determining suitable machine learning classifier technique for prediction of malaria incidents attributed to climate of Odisha [J]. Int J Environ Health Res, 2022, 32(8): 1716-1732.

[222] TAI K Y, DHALIWAL J. Machine learning model for malaria risk prediction based on mutation location of large-scale genetic variation data [J]. JOURNAL OF BIG DATA, 2022, 9(1).

[223] VAVILALA H, YALADANDA N, KONDETI P K, et al. Weather integrated malaria prediction system using Bayesian structural time series model for northeast states of India [J]. ENVIRONMENTAL SCIENCE AND POLLUTION RESEARCH, 2022.

[224] ZHAO Z H, LI S C, LU Y L. MATHEMATICAL MODELS FOR THE TRANSMISSION OF MALARIA WITH SEASONALITY AND IVERMECTIN [J]. ELECTRONIC JOURNAL OF DIFFERENTIAL EQUATIONS, 2022, 2022(28).

[225] IMAI N, WHITE M T, GHANI A C, et al. Transmission and Control of Plasmodium knowlesi: A Mathematical Modelling Study [J]. PLoS Negl Trop Dis, 2014, 8(7).

[226] HOD R, MOKHTAR S A, MUHARAM F M, et al. Developing a Predictive Model for Plasmodium knowlesi-Susceptible Areas in Malaysia Using Geospatial Data and Artificial Neural Networks [J]. Asia Pac J Public Health, 2022, 34(2): 182-190.

[227] JONES A E, MORSE A P J J O C. Application and Validation of a Seasonal Ensemble Prediction System Using a Dynamic Malaria Model [J]. Journal of Climate, 2010, 23(15): 4202-4215.

[228] Garcia KKS, Abrahão AA, Oliveira AFM, Henriques KMD, de Pina-Costa A, Siqueira AM, Ramalho WM. Malaria time series in the extra-Amazon region of Brazil: epidemiological scenario and a two-year prediction model. Malar J. 2022 May 31;21(1):157.

[229] Zhou SS, Huang F, Wang JJ, Zhang SS, Su YP, Tang LH. Geographical, meteorological and vectorial factors related to malaria re-emergence in Huang-Huai River of central China. Malar J. 2010 Nov 24;9:337.

[230] Gao HW, Wang LP, Liang S, Liu YX, Tong SL, Wang JJ, Li YP, Wang XF, Yang H, Ma JQ, Fang LQ, Cao WC. Change in rainfall drives malaria re-emergence in Anhui Province, China. PLoS One. 2012;7(8):e43686.

[231] Chen Z, Shi L, Zhou XN, Xia ZG, Bergquist R, Jiang QW. Elimination of malaria due to Plasmodium vivax in central part of the People's Republic of China: analysis and prediction based on modelling. Geospat Health. 2014 Nov;9(1):169-77.

[232] Buczak AL, Baugher B, Guven E, Ramac-Thomas LC, Elbert Y, Babin SM, Lewis SH. Fuzzy association rule mining and classification for the prediction of malaria in South Korea. BMC Med Inform Decis Mak. 2015 Jun 18;15:47.

[233] Xiaohuan WANG. Establishment of re-epidemic risk index system and synthetical evaluation in the elimination of malaria,Fujian Province,China. Fujian Medical University. 2015.

[234] Xiaohuan WANG, Shanying ZHANG. Establishment of re-epidemic risk index system of the elimination of malaria in Fujian Province. Chinese Journal of Zoonoses. 2015,31(11):1081-1085.

[235] Ranjbar M, Shoghli A, Kolifarhood G, Tabatabaei SM, Amlashi M, Mohammadi M. Predicting factors for malaria re-introduction: an applied model in an elimination setting to prevent malaria outbreaks. Malar J. 2016 Mar 2;15:138.

[236] Shouqin YIN. Risk Assessment for Malaria Transmission in the Border Area of Yunnan Province. Chinese Central for Disease Control and Prevention.2016.

[237] Shouqin YIN, Shang XIA, Xingwu ZHOU, et al. Risk Assessment for Malaria Transmission in the Border Area of Yunnan Province. Chin J Parasitol Parasit Dis. 2016,34(03):255-260.

[238] Lei LEI. Risk Assessment of the Secondary Transmission by Imported Malaria in Jiangxi Province. Chinese Central for Disease Control and Prevention.2017.

[239] Lei LEI, Zhigui XIA, Zhihong LI, et al. Risk assessment of secondary transmission induced by imported malaria in Jiangxi Province. Chin J Schisto Control. 2017,29(02):182-187.

[240] Chuang TW, Soble A, Ntshalintshali N, Mkhonta N, Seyama E, Mthethwa S, Pindolia D, Kunene S. Assessment of climate-driven variations in malaria incidence in Swaziland: toward malaria elimination. Malar J. 2017 Jun 1;16(1):232.

[241] Chunyan LIU, Shangfeng TANG, Mengxue LI, et al. Establishment of an index system for malaria risk assessment in China. Chin J Public Health. 2017,33(04):524-527.

[242] Tianmu CHEN. Study on indicators and modelling of risk assessment on malaria re-establishment. Chinese Central for Disease Control and Prevention. 2018.

[243] Lei L, Richards JS, Li ZH, Gong YF, Zhang SZ, Xiao N. A framework for assessing local transmission risk of imported malaria cases. Infect Dis Poverty. 2019 Jun 7;8(1):43.

[244] Zhao X, Thanapongtharm W, Lawawirojwong S, Wei C, Tang Y, Zhou Y, Sun X, Cui L, Sattabongkot J, Kaewkungwal J. Malaria Risk Map Using Spatial Multi-Criteria Decision Analysis along Yunnan Border During the Pre-elimination Period. Am J Trop Med Hyg. 2020 Aug;103(2):793-809.

[245] Guoqun LI, Yu LIAO, Xiuqin HONG, et al. Risk prediction of imported malaria re-transmission in Changsha based on SEIR-SEI model. Modern Preventive Medicine. 2021,48(11):1921-1929.

[246] Qian LI. Risk analysis of preventing re-transmission after malaria elimination in Fujian Province. Fujian Medical University. 2021.

[247] Xiaotong MO. Study on risk assessment on malaria importation and re-establishment in China during malaria elimination. Chinese Central for Disease Control and Prevention. 2021

[248] Xiaotong MO, Shang XIA, Lin AI, et al. Study on a framework for risk assessment of imported malaria in China during malaria elimination. China Tropical Medicine. 2021,21(6):505-511.

[249] Linard Catherine, Nicolas Ponçon, et al. A multi-agent simulation to assess the risk of malaria re-emergence in southern France. Ecological Modelling. 2008 220: 160-174.

[250] Linard C, Ponçon N, Fontenille D, Lambin EF. Risk of malaria reemergence in southern France: testing scenarios with a multiagent simulation model. Ecohealth. 2009 Mar;6(1):135-47.

[251] Martineau P, Behera SK, Nonaka M, Jayanthi R, Ikeda T, Minakawa N, Kruger P, Mabunda QE. Predicting malaria outbreaks from sea surface temperature variability up to 9 months ahead in Limpopo, South Africa, using machine learning. Front Public Health. 2022 Aug 25;10:962377.

[252] Salahi-Moghaddam A, Turki H, Yeryan M, Fuentes MV. Spatio-temporal Prediction of the Malaria Transmission Risk in Minab District (Hormozgan Province, Southern Iran). Acta Parasitol. 2022 Dec;67(4):1500-1513.

**2.** Prediction of vector density or dynamics**^[1-33]^:**

[1] REGION S. PREDICTIVE HABITAT MODELLING FOR FLORA AND FAUNA SPECIES IN THE [J].

[2] REJMANKOVA E, ROBERTS D R, PAWLEY A, et al. Predictions of adult Anopheles albimanus densities in villages based on distances to remotely sensed larval habitats [J]. Am J Trop Med Hyg, 1995, 53(5): 482-488.

[3] SMITH T, CHARLWOOD J D, TAKKEN W, et al. Mapping the densities of malaria vectors within a single village [J]. Acta Trop, 1995, 59(1): 1-18.

[4] ROBERTS D R, PARIS J F, MANGUIN S, et al. Predictions of Malaria Vector Distribution in Belize Based on Multispectral Satellite Data [J]. 1996, 54(3): 304-308.

[5] SHAMAN J, STIEGLITZ M, STARK C, et al. Using a dynamic hydrology model to predict mosquito abundances in flood and swamp water [J]. Emerg Infect Dis, 2002, 8(1): 6-13.

[6] BIAN L, LI L, YAN G J M S, et al. Combining Global and Local Estimates for Spatial Distribution of Mosquito Larval Habitats [J]. 2006, 43(2): 128-141.

[7] SCHRöDER W, SCHMIDT G, BAST H, et al. Pilot-study on GIS-based risk modelling of a climate warming induced tertian malaria outbreak in Lower Saxony (Germany) [J]. Environ Monit Assess, 2007, 133(1-3): 483-493.

[8] SWEENEY A W, BEEBE N W, COOPER R D J E M. Analysis of environmental factors influencing the range of anopheline mosquitoes in northern Australia using a genetic algorithm and data mining methods [J]. 2007, 203(3-4): 375-386.

[9] BANERJEE A K, KIRAN K, MURTY U S, et al. Classification and identification of mosquito species using artificial neural networks [J]. Comput Biol Chem, 2008, 32(6): 442-447.

[10] BROWN H E, DIUK-WASSER M A, GUAN Y, et al. Comparison of three satellite sensors at three spatial scales to predict larval mosquito presence in Connecticut wetlands [J]. 2008, 112(5): 2301-2308.

[11] FOLEY D H, KLEIN T A, KIM H C, et al. Malaria risk assessment for the Republic of Korea based on models of mosquito distribution [J]. US Army Med Dep J, 2008: 46-53.

[12] JACOB B J, GU W, CAAMANO E X, et al. Developing operational algorithms using linear and non-linear squares estimation in Python for the identification of Culex pipiens and Culex restuans in a mosquito abatement district (Cook County, Illinois, USA) [J]. Geospat Health, 2009, 3(2): 157-176.

[13] KULKARNI M A, DESROCHERS R E, KERR J T. High resolution niche models of malaria vectors in northern Tanzania: a new capacity to predict malaria risk? [J]. PLoS One, 2010, 5(2): e9396.

[14] LI L, BIAN L, YAKOB L, et al. Analysing the generality of spatially predictive mosquito habitat models [J]. Acta Trop, 2011, 119(1): 30-37.

[15] CHUANG T W, HENEBRY G M, KIMBALL J S, et al. Satellite Microwave Remote Sensing for Environmental Modeling of Mosquito Population Dynamics [J]. Remote Sens Environ, 2012, 125: 147-156.

[16] NMOR J C, SUNAHARA T, GOTO K, et al. Topographic models for predicting malaria vector breeding habitats: potential tools for vector control managers [J]. Parasit Vectors, 2013, 6: 14.

[17] OLUWAGBEMI O O, FORNADEL C M, ADEBIYI E F, et al. ANOSPEX: a stochastic, spatially explicit model for studying Anopheles metapopulation dynamics [J]. PLoS One, 2013, 8(7): e68040.

[18] MOIROUX N, DJèNONTIN A, BIO-BANGANA A S, et al. Spatio-temporal analysis of abundances of three malaria vector species in southern Benin using zero-truncated models [J]. Parasit Vectors, 2014, 7: 103.

[19] MA A M, WANG J F, WANG D Q, et al. Prediction of potential distribution of Anopheles sinensis in China based on MaxEnt [J]. Chinese Journal of Vector Biology and Control, 2014, 25(5): 6.

[20] IBAñEZ-JUSTICIA A, CIANCI D. Modelling the spatial distribution of the nuisance mosquito species Anopheles plumbeus (Diptera: Culicidae) in the Netherlands [J]. Parasit Vectors, 2015, 8: 258.

[21] ADDE A, DUSFOUR I, ROUX E, et al. Anopheles fauna of coastal Cayenne, French Guiana: modelling and mapping of species presence using remotely sensed land cover data [J]. Mem Inst Oswaldo Cruz, 2016, 111(12): 750-756.

[22] ADDE A, ROUX E, MANGEAS M, et al. Dynamical Mapping of Anopheles darlingi Densities in a Residual Malaria Transmission Area of French Guiana by Using Remote Sensing and Meteorological Data [J]. PLoS One, 2016, 11(10): e0164685.

[23] SINKA M E, GOLDING N, MASSEY N C, et al. Modelling the relative abundance of the primary African vectors of malaria before and after the implementation of indoor, insecticide-based vector control [J]. Malar J, 2016, 15: 142.

[24] CARVALHO B M, RANGEL E F, VALE M M. Evaluation of the impacts of climate change on disease vectors through ecological niche modelling [J]. Bull Entomol Res, 2017, 107(4): 419-430.

[25] PAKDAD K, HANAFI-BOJD A A, VATANDOOST H, et al. Predicting the potential distribution of main malaria vectors Anopheles stephensi, An. culicifacies s.l. and An. fluviatilis s.l. in Iran based on maximum entropy model [J]. Acta Trop, 2017, 169: 93-99.

[26] MALAHLELA O E, ADJORLOLO C, OLWOCH J M, et al. Integrating geostatistics and remote sensing for mapping the spatial distribution of cattle hoofprints in relation to malaria vector control [J]. 2019, 40(15-16): 5917-5937.

[27] FRAKE A N, PETER B G, WALKER E D, et al. Leveraging big data for public health: Mapping malaria vector suitability in Malawi with Google Earth Engine [J]. PLoS One, 2020, 15(8): e0235697.

[28] RAHMAN M S, PIENTONG C, ZAFAR S, et al. Mapping the spatial distribution of the dengue vector Aedes aegypti and predicting its abundance in northeastern Thailand using machine-learning approach [J]. One Health, 2021, 13: 100358.

[29] RHODES C G, LOAIZA J R, ROMERO L M, et al. Anopheles albimanus (Diptera: Culicidae) Ensemble Distribution Modeling: Applications for Malaria Elimination [J]. Insects, 2022, 13(3).

[30] LUNDE T M, KORECHA D, LOHA E, et al. A dynamic model of some malaria-transmitting anopheline mosquitoes of the Afrotropical region. I. Model description and sensitivity analysis [J]. 2013, 12.

[31] MOUA Y, ROUX E, GIROD R, et al. Distribution of the Habitat Suitability of the Main Malaria Vector in French Guiana Using Maximum Entropy Modeling [J]. J Med Entomol, 2017, 54(3): 606-621.

[32] SANTOSH T, RAMESH D, REDDY D. LSTM based prediction of malaria abundances using big data [J]. Comput Biol Med, 2020, 124: 103859.

[33] HOLY M, SCHMIDT G, SCHRODER W. Potential malaria outbreak in Germany due to climate warming: risk modelling based on temperature measurements and regional climate models [J]. ENVIRONMENTAL SCIENCE AND POLLUTION RESEARCH, 2011, 18(3): 428-435.

[34] Tong YX, Xia ZG, Wang QY, Xu N, Jiang HL, Wang ZZ, Xiong Y, Yin JF, Huang JH, Jiang F, Chen Y, Jiang QW, Zhou YB. Prediction of the Risk Distributions for Anopheles sinensis, a Vector for Malaria in Shanghai, China. Am J Trop Med Hyg. 2023 Jan 23;108(3):599-608.

**3.** Studies was not in humans**^[1-30]^:**

[1] WATKINS W M, MBERU E K, WINSTANLEY P A, et al. The efficacy of antifolate antimalarial combinations in Africa: A predictive model based on pharmacodynamic and pharmacokinetic analyses [J]. Parasitol Today, 1997, 13(12): 459-464.

[2] AVERY M A, ALVIM-GASTON M, RODRIGUES C R, et al. Structure-activity relationships of the antimalarial agent artemisinin. 6. The development of predictive in vitro potency models using CoMFA and HQSAR methodologies [J]. J Med Chem, 2002, 45(2): 292-303.

[3] HOSHEN M B, STEIN W D, GINSBURG H. Mathematical modelling of malaria chemotherapy: combining artesunate and mefloquine [J]. Parasitology, 2002, 124: 9-15.

[4] PRADO-PRADO F J, GONZALEZ-DIAZ H, DE LA VEGA O M, et al. Unified QSAR approach to antimicrobials. Part 3: First multi-tasking QSAR model for Input-Coded prediction, structural back-projection, and complex networks clustering of antiprotozoal compounds [J]. Bioorg Med Chem, 2008, 16(11): 5871-5880.

[5] LI Y, LIU Y Y, GAO F, et al. Predictive model for flavonoid inhibition of Plasmodium falciparum [J]. Computers and Applied Chemistry, 2010, 27(4): 485-491.

[6] NAIK P K, SRIVASTAVA M, BAJAJ P, et al. The binding modes and binding affinities of artemisinin derivatives with Plasmodium falciparum Ca2+-ATPase (PfATP6) [J]. J Mol Model, 2011, 17(2): 333-357.

[7] GOGOI D, DUTTA P, YADAV R N S. 3D QSAR modeling of 4-nerolidylcatechol derivatives and virtual screening for identification of potent plasmodium inhibitor [J]. BANGLADESH JOURNAL OF PHARMACOLOGY, 2014, 9(3): 317-321.

[8] MASAND V H, TOROPOV A A, TOROPOVA A P, et al. QSAR Models for Anti-Malarial Activity of 4-Aminoquinolines [J]. Curr Comput Aided Drug Des, 2014, 10(1): 75-82.

[9] SHARMA M C, SHARMA S, SHARMA P, et al. Pharmacophore and QSAR modeling of some structurally diverse azaaurones derivatives as anti-malarial activity [J]. Med Chem Res, 2014, 23(1): 181-198.

[10] YADAV M K, SINGH A, SWATI D. A Knowledge-Based Approach for Identification of Drugs Against Vivapain-2 Protein of Plasmodium vivax Through Pharmacophore-Based Virtual Screening with Comparative Modelling [J]. Appl Biochem Biotechnol, 2014, 173(8): 2174-2188.

[11] KHATRI N, DUTT R, MADAN A K. Role of moving average analysis for development of multi-target (Q)SAR models [J]. Mini Rev Med Chem, 2015, 15(8): 659-676.

[12] KUMARI M, CHANDRA S. In silico prediction of anti-malarial hit molecules based on machine learning methods [J]. Int J Comput Biol Drug Des, 2015, 8(1): 40-53.

[13] SINGH S. Computational design and chemometric QSAR modeling of Plasmodium falciparum carbonic anhydrase inhibitors [J]. Bioorg Med Chem Lett, 2015, 25(1): 133-141.

[14] POONGAVANAM V, KONGSTED J. Binding affinity models for Falcipain inhibition based on the Linear Interaction Energy method [J]. J Mol Graph Model, 2016, 70: 236-245.

[15] BHARTI D R, LYNN A M. QSAR based predictive modeling for anti-malarial molecules [J]. Bioinformation, 2017, 13(5): 154-159.

[16] CAO P X, KLONIS N, ZALOUMIS S, et al. A Dynamic Stress Model Explains the Delayed Drug Effect in Artemisinin Treatment of Plasmodium falciparum [J]. Antimicrob Agents Chemother, 2017, 61(12).

[17] HUY N T, CHI P L, NAGAI J, et al. High-Throughput Screening and Prediction Model Building for Novel Hemozoin Inhibitors Using Physicochemical Properties [J]. Antimicrob Agents Chemother, 2017, 61(2).

[18] NATTEE C, KHAMSEMANAN N, LAWTRAKUL L, et al. A novel prediction approach for antimalarial activities of Trimethoprim, Pyrimethamine, and Cycloguanil analogues using extremely randomized trees [J]. J Mol Graph Model, 2017, 71: 13-27.

[19] EGIEYEH S, SYCE J, MALAN S F, et al. Predictive classifier models built from natural products with antimalarial bioactivity using machine learning approach [J]. PLoS One, 2018, 13(9).

[20] KHAMSEMANAN N, NATTEE C, LAWTRAKUL L, et al. PREDICTING INHIBITION CONSTANTS OF ANTIMALARIAL DRUG COMPOUNDS USING SUPPORT VECTOR REGRESSION [J]. JOURNAL OF NONLINEAR AND CONVEX ANALYSIS, 2018, 19(9): 1555-1562.

[21] MASON D J, EASTMAN R T, LEWIS R P I, et al. Using Machine Learning to Predict Synergistic Antimalarial Compound Combinations With Novel Structures [J]. Front Pharmacol, 2018, 9.

[22] SHAYAAN A, ILANCHEZIAN I, RAO S, et al. Prediction of Malaria Vaccination Outcomes from Gene Expression Data; proceedings of the PROCEEDINGS OF THE 12TH INTERNATIONAL JOINT CONFERENCE ON BIOMEDICAL ENGINEERING SYSTEMS AND TECHNOLOGIES, VOL 3 (BIOINFORMATICS), F 2019, 2019 [C].

[23] SYED A H, KHAN T. A supervised classifier based chemoinformatics model to predict inhibitors essential for sexual reproduction and transmission of the P. falciparum parasite into mosquitoes [J]. INTERNATIONAL JOURNAL OF ADVANCED AND APPLIED SCIENCES, 2019, 6(10): 62-72.

[24] YOUSEFINEJAD S, MAHBOUBIFAR M, ESKANDARI R. Quantitative structure-activity relationship to predict the anti-malarial activity in a set of new imidazolopiperazines based on artificial neural networks [J]. Malar J, 2019, 18(1).

[25] MSWAHILI M E, MARTIN G L, WOO J, et al. Antimalarial Drug Predictions Using Molecular Descriptors and Machine Learning against Plasmodium Falciparum [J]. BIOMOLECULES, 2021, 11(12).

[26] NGUYEN P T V, DAT T V, MIZUKAMI S, et al. 2D-quantitative structure-activity relationships model using PLS method for anti-malarial activities of anti-haemozoin compounds [J]. Malar J, 2021, 20(1).

[27] HESPING E, CHUA M J, PFLIEGER M, et al. QSAR Classification Models for Prediction of Hydroxamate Histone Deacetylase Inhibitor Activity against Malaria Parasites [J]. ACS INFECTIOUS DISEASES, 2022, 8(1): 106-117.

[28] PROIETTI C, KRAUSE L, TRIEU A, et al. Immune Signature Against Plasmodium falciparum Antigens Predicts Clinical Immunity in Distinct Malaria Endemic Communities [J]. Mol Cell Proteomics, 2020, 19(1): 101-113.

[29] PANDEY S, NANDA S, VUTHA A, et al. Modeling the impact of biolarvicides on malaria transmission [J]. J Theor Biol, 2018, 454: 396-409.

[30] SYED A H, KHAN T J I J O A, SCIENCES A. A supervised classifier based chemoinformatics model to predict inhibitors essential for sexual reproduction and transmission of the P. falciparum parasite into mosquitoes [J]. 2019, 6(10): 62-72.

**4.** No prediction models established**^[1-12]^：**

[1] GERARDIN P, ROGIER C, LETEURTRE S, et al. Evaluation of Pediatric Risk of Mortality (PRISM) scoring in African children with falciparum malaria [J]. Pediatr Crit Care Med, 2006, 7(1): 45-47.

[2] NOOR A M, UUSIKU P, KAMWI R N, et al. The receptive versus current risks of Plasmodium falciparum transmission in Northern Namibia: implications for elimination [J]. BMC Infect Dis, 2013, 13.

[3] OCAMPO A J, CHUNARA R, BROWNSTEIN J S. Using search queries for malaria surveillance, Thailand [J]. Malar J, 2013, 12.

[4] KWAK J, NOH H, KIM S, et al. Future climate data from RCP 4.5 and occurrence of malaria in Korea [J]. Int J Environ Res Public Health, 2014, 11(10): 10587-10605.

[5] BBOSA F, WESONGA R, JEHOPIO P. Clinical malaria diagnosis: rule-based classification statistical prototype [J]. SPRINGERPLUS, 2016, 5.

[6] BENNETT A, YUKICH J, MILLER J M, et al. The relative contribution of climate variability and vector control coverage to changes in malaria parasite prevalence in Zambia 2006-2012 [J]. PARASITES & VECTORS, 2016, 9.

[7] VAN WOLFSWINKEL M E, KOOPMANS L C, HESSELINK D A, et al. Neutrophil gelatinase-associated lipocalin (NGAL) predicts the occurrence of malaria-induced acute kidney injury [J]. Malar J, 2016, 15.

[8] WANG X L, YANG B, HUANG J, et al. IASM: A System for the Intelligent Active Surveillance of Malaria [J]. Comput Math Methods Med, 2016, 2016.

[9] XIANG-HUA M, YAN D, YING D, et al. Time characteristics of imported malaria cases in Yunnan Province [J]. Chinese journal of schistosomiasis control, 2017, 29(4): 445-448.

[10] SOW B, MUKHTAR H, AHMAD H F, et al. Assessing the relative importance of social determinants of health in malaria and anemia classi?cation based on machine learning techniques [J]. INFORMATICS FOR HEALTH & SOCIAL CARE, 2020, 45(3): 229-241.

[11] CHAUDHURY S, YU C G, LIU R F, et al. Wearables Detect Malaria Early in a Controlled Human-Infection Study [J]. IEEE Trans Biomed Eng, 2022, 69(6): 2119-2129.

[12] HARRIS M J, HAY S I, DRAKE J M. Early warning signals of malaria resurgence in Kericho, Kenya [J]. Biol Lett, 2020, 16(3): 20190713.

[13] Jeevatharan H, Wickremasinghe R. Susceptibility to malaria during the prevention of re-establishment phase in Sri Lanka. Malar J. 2022 Mar 27;21(1):108.

**5.**Not focused on malaria**^[1-6]^：**

[1] KHATRI N, DUTT R, MADAN A K. Role of moving average analysis for development of multi-target (Q)SAR models [J]. Mini Rev Med Chem, 2015, 15(8): 659-676.

[2] GANDON S, DAY T, METCALF C J E, et al. Forecasting Epidemiological and Evolutionary Dynamics of Infectious Diseases [J]. Trends Ecol Evol, 2016, 31(10): 776-788.

[3] SONG X, XIAO J, DENG J, et al. Time series analysis of influenza incidence in Chinese provinces from 2004 to 2011 [J]. Medicine, 2016, 95(26).

[4] CHEN Y, CHU C W, CHEN M I C, et al. The utility of LASSO-based models for real time forecasts of endemic infectious diseases: A cross country comparison [J]. J Biomed Inform, 2018, 81: 16-30.

[5] GIORGI E, DIGGLE P J, SNOW R W, et al. Geostatistical Methods for Disease Mapping and Visualisation Using Data from Spatio-temporally Referenced Prevalence Surveys [J]. INTERNATIONAL STATISTICAL REVIEW, 2018, 86(3): 571-597.

[6] EJIGU B A, WENCHEKO E, MORAGA P, et al. Geostatistical methods for modelling non-stationary patterns in disease risk [J]. SPATIAL STATISTICS, 2020, 35.

[7] Javaid M, Sarfraz MS, Aftab MU, Zaman QU, Rauf HT, Alnowibet KA. WebGIS-Based Real-Time Surveillance and Response System for Vector-Borne Infectious Diseases. Int J Environ Res Public Health. 2023 Feb 20;20(4):3740.

6. Study location was not specified**^[1]^：**

[1] EZE P U, ASOGWA C O. Deep Machine Learning Model Trade-Offs for Malaria Elimination in Resource-Constrained Locations [J]. Bioengineering (Basel), 2021, 8(11).

**7.** Not full text publication**^[1]^:**

[1] RODHAIN F, CHARMOT G. ASSESSMENT OF THE RISK OF MALARIA REINTRODUCTION IN FRANCE [J]. Med Mal Infect, 1982, 12(4): 231-236.

# Appendix 5

**Table S1. Limitations of the included studies (n=10)**

| **No** | **Author, year** | **The limitations of the model** |
| --- | --- | --- |
| **1** | **Schöder et al, 2008** | 1.The relationship between climate and transmission potential of malaria is still only partly understood.  2.Certain variables which can play a significant role in the complex dynamic process of disease spread were excluded.  3.The impact of climate change poses enormous challenges because of the considerable amount of complexity and uncertainty. |
| **2** | **Linard et al., 2009** | 1.The model is very sensitive to the mortality and reproduction rates of mosquitoes. The estimation of these two parameters should therefore deserve more attention, even though they are difficult to measure in natural conditions.  2.we considered the entire population of mosquitoes on one pixel as one population unit. We thus overlooked details of within-patch dynamics.  3.Some external factors such as the wind and relative humidity were not explicitly taken into account in the model. There is a lack of quantitative data on their impact on mosquito behavior. |
| **5** | **Sainz-Elipe et al., 2010** | Not reported |
| **8** | **Romi et al., 2012** | Not reported |
| **9** | **Sudre et al., 2013** | A major limitation is that it considers only environmental suitability for transmission, not risk for transmission perse. It did not highlight areas of northern Greece previously associated with malaria as being particularly suitable for transmission. |
| **18** | **Pergantas et al., 2017** | 1. The selected regions were based on field studies and were not selected within a large randomized experiment. Therefore these results are representative of the studied (and similar) region.   2. Weekly—and not daily—samples were collected so these numbers are subject to sampling bias.  3. While validation against an external/ independent source of evidence is desirable whenever an analysis with potential for policy action is performed, this seemed nearly impossible in our case since we are essentially looking at counterfactual scenarios because what actually happened is the result of different interventions of distinct nature for each area.  4. It had limited data on the mobility patterns of potential hosts. |
| **26** | **Kamana et al., 2022** | 1.The LSTMSeq2Seq takes more time for training than other employed deep learning models. To train the LSTMSeq2Seq from scratch for all 31 provinces takes 2 weeks for four types of Plasmodium used in our study, whereas other models take a few hours to days to train them using malaria cases and data of meteorological variables. However, the impact model is not significant in provinces with fewer malaria cases.  2.We could not obtain accurate predictions in some provinces by using any model in this study, probably because we failed to get other relevant potential non-climatic factors. |
| **27** | **Lan et al., 2022** | Not reported |
| **28** | **Li et al., 2022** | Not reported |
|  | **Liu et al., 2023** | Not reported |

# Appendix 6

**Table S2.** Quality assessment of included studies using the PROBAST scale for predictive modeling studies

| Author  year | Risk of bias | | | |  | Applicability | | |  | Overall | |
| --- | --- | --- | --- | --- | --- | --- | --- | --- | --- | --- | --- |
|  | Participants | Predictors | Outcome | Analysis |  | Participants | Predictors | Outcome |  | Risk of bias | Applicability |
| Schöder and Schmidt 2008 | high | low | unclear | high |  | low | high | low |  | high | high |
| Linard et al.,  2009 | low | high | unclear | high |  | low | low | low |  | high | low |
| Sainz-Elipe et al.,  2010 | low | low | unclear | high |  | low | low | low |  | high | low |
| Romi et al.,2012 | low | high | unclear | high |  | low | low | low |  | high | low |
| Sudre et al.,2013 | high | low | unclear | high |  | high | low | low |  | high | high |
| Pergantas et al.,  2017 | low | low | unclear | high |  | low | low | low |  | high | low |
| Kamana et al.,  2022 | low | high | high | high |  | low | unclear | low |  | high | unclear |
| Lan et al.,2022 | unclear | unclear | high | high |  | unclear | low | unclear |  | high | low |
| Li et al.,2022 | low | low | high | high |  | low | low | low |  | high | low |
| Liu et al, 2023 | high | unclear | unclear | high |  | low | unclear | low |  | high | unclear |

# Figure S1. Risk of bias and applicability assessment according to the Prediction model Risk of Bias Assessment Tool (PROBAST).

#
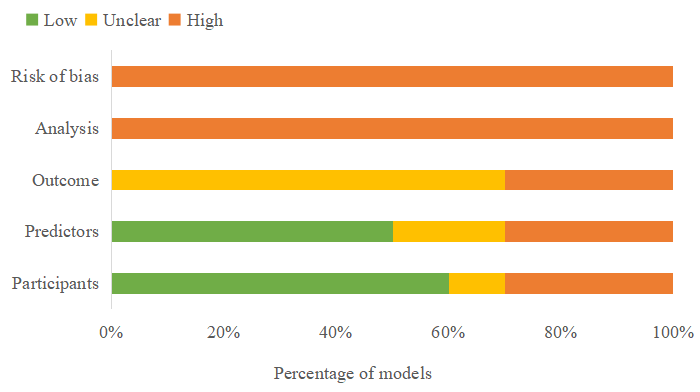

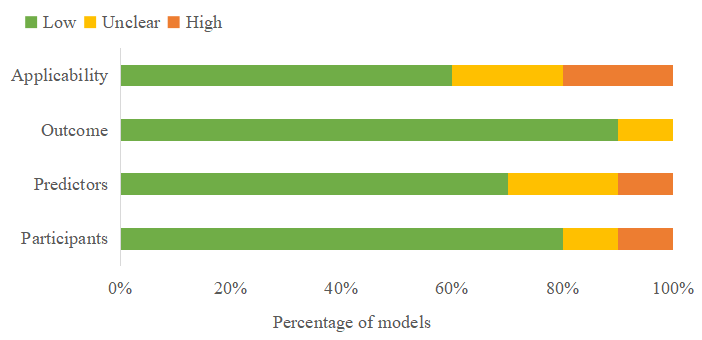


# Appendix 7

## **Table S3.** The aNOS for assessing the quality of cross sectional studies

| Author  year | Selection | | | |  | Comparability |  | Outcome | | NOS scale  (max. 10) |
| --- | --- | --- | --- | --- | --- | --- | --- | --- | --- | --- |
|  | Representa-  tiveness of the sample  ness of the exposed cohort | Sample size | Non-  respondents | Ascertainment of the exposure |  | Comparability of studies on the basis of the design or analysis |  | Assessment of the outcome | Statistical test |  |
| Schöder and Schmidt 2008 | a | a | a | b |  | a |  | b | b | 7 |
| Linard et al.,2009 | a | a | a | b |  | a |  | a | b | 7 |
| Sainz-Elipe et al., 2010 | a | a | a | b |  | a |  | a | b | 7 |
| Romi et al.,2012 | a | a | a | b |  | a |  | a | b | 7 |
| Sudre et al.,2013 | a | a | a | a |  | a |  | b | b | 8 |
| Pergantas et al.,2017 | a | a | a | b |  | a |  | a | b | 7 |
| Kamana et al., 2022 (+4) | a | a | a | b |  | a |  | b | b | 7 |
| Lan et al.,2022 | c | b | c | c |  | a |  | d | a | 3 |
| Li et al.,2022 | b | a | a | b |  | a |  | b | a | 8 |
| Liu et al.,2022 | b | a | a | a |  | a |  | d | a | 7 |
